# Effect of fecal microbiota transplantation on neurological restoration in a spinal cord injury mouse model: involvement of brain-gut axis

**DOI:** 10.1186/s40168-021-01007-y

**Published:** 2021-03-07

**Authors:** Yingli Jing, Yan Yu, Fan Bai, Limiao Wang, Degang Yang, Chao Zhang, Chuan Qin, Mingliang Yang, Dong Zhang, Yanbing Zhu, Jianjun Li, Zhiguo Chen

**Affiliations:** 1 China Rehabilitation Science Institute, China Rehabilitation Research Center, Beijing Key Laboratory of Neural Injury and Rehabilitation, and School of Rehabilitation Medicine, Capital Medical University, Beijing, China; 2 Center of Neural Injury and Repair, Beijing Institute for Brain Disorders, Beijing, China; 3 Immunology Research Center for Oral and Systemic Health, Beijing Friendship Hospital, Capital Medical University, Beijing, China; 4 Beijing Key Laboratory of Tolerance Induction and Organ Protection in Transplantation, Beijing, China; 5 Beijing Clinical Research Institute, Beijing, China; 6 Cell Therapy Center, Beijing Institute of Geriatrics, Xuanwu Hospital Capital Medical University, National Clinical Research Center for Geriatric Diseases, and Key Laboratory of Neurodegenerative Diseases, Ministry of Education, Beijing, China

**Keywords:** Fecal microbiota transplantation, Neurological function, GI function, Gut microbiota, Neuroinflammation

## Abstract

**Background:**

Spinal cord injury (SCI) patients display disruption of gut microbiome, and gut dysbiosis exacerbate neurological impairment in SCI models. Cumulative data support an important role of gut microbiome in SCI. Here, we investigated the hypothesis that fecal microbiota transplantation (FMT) from healthy uninjured mice into SCI mice may exert a neuroprotective effect.

**Results:**

FMT facilitated functional recovery, promoted neuronal axonal regeneration, improved animal weight gain and metabolic profiling, and enhanced intestinal barrier integrity and GI motility in SCI mice. High-throughput sequencing revealed that levels of phylum *Firmicutes*, family *Christensenellaceae*, and genus *Butyricimonas* were reduced in fecal samples of SCI mice, and FMT remarkably reshaped gut microbiome. Also, FMT-treated SCI mice showed increased amount of fecal short-chain fatty acids (SCFAs), which correlated with alteration of intestinal permeability and locomotor recovery. Furthermore, FMT downregulated IL-1β/NF-κB signaling in spinal cord and NF-κB signaling in gut following SCI.

**Conclusion:**

Our study demonstrates that reprogramming of gut microbiota by FMT improves locomotor and GI functions in SCI mice, possibly through the anti-inflammatory functions of SCFAs.

Video Abstract

**Supplementary Information:**

The online version contains supplementary material available at 10.1186/s40168-021-01007-y.

## Background

The gastrointestinal (GI) tract has the unique property of harboring numerous microbes within the lumen, with the cell number of bacteria roughly the same as that of human cells [1]. The commensal microbiome affects intestinal physiology and modulates functions of the immune and endocrine systems of host [2–5]. Emerging evidence indicates that the composition and metabolites of microbiome not only regulate normal development of the central nervous system (CNS) such as formation of blood-brain barrier, myelination, neurogenesis, and microglia maturation [6–8], but also contribute to the onset and/or progression of neurological diseases including Alzheimer’s disease, Parkinson’s disease, stroke, depression, anxiety, and autism [9–14].

Traumatic spinal cord injury (SCI) causes neurological impairment and secondary complications [15], such as colorectal, bladder, and sexual dysfunctions, among which recovery of bowel function is even prioritized above the ability to walk to some researchers [16–18]. GI dysfunction often manifests as diminished colonic transit, constipation, evacuation dyssynergia, and overflow incontinence, which occur frequently in SCI and may aggravate neurological impairment [19]. Most recently, many lines of evidence demonstrated that aberrant gut microbial community is involved in the pathogenesis and clinical symptoms of SCI [20–23]. Moreover, a strong correlation between the relative abundance of *Clostridiales* and *Anaeroplasmatales* and open-field locomotor has been observed in SCI mice, indicating that the proportion of these bacteria might predict the size of functional recovery [22]. Recent investigations demonstrated that induced gut dysbiosis exacerbates lesion pathology and impairs functional recovery after SCI, whereas remodeling gut microbes is beneficial to locomotor recovery following injury [22, 24]. These studies have emphasized a strong correlation between gut dysbiosis and SCI and further implied the significance of gut microbiome in neurological regulation.

Microbiota-targeted technique (e.g., FMT) might be useful for treatment of different CNS diseases via remodeling gut microbiota, yet the mechanisms of FMT in different CNS diseases have not been well understood. Currently, the research field on microbiota-gut-brain axis is gaining more attention, and the advance of the field has shed more light on the physiological and pathologic basis of neural restoration. Microbiota and brain communicate via various routes including the immune system and nervous system, which may involve microbial metabolites such as short-chain fatty acids (SCFAs) [25]. In APP/PS1 transgenic mice, FMT from wild-type mice alleviates Alzheimer’s disease-like pathogenesis by reduction of COX-2 and CD11b levels, and by alteration of gut microbiota and SCFAs [26]. In an MPTP-induced Parkinson’s disease mouse model, FMT from healthy control mice exerts a neuroprotective effect through inhibition of glia cell activation and modulating expression of fecal SCFAs [27]. In two different germ-free multiple sclerosis (MS), mouse models induced either by expressing a transgenic myelin oligodendrocyte glycoprotein (MOG)-specific T cell receptor in most CD4+ T cells [28] or by immunization with a fragment of MOG (MOG 35–55) [29], colonization of the germ-free mice with MS patient- vs. healthy donor-derived microbiota can significantly exacerbate experimental autoimmune encephalomyelitis symptoms. In this study, we hypothesize that FMT from healthy uninjured mice into SCI mice may remodel gut microbiome and be neuroprotective in SCI.

## Materials

### Animals

Adult female C57BL/6N (18–22 g) mice were purchased from the Center of Experimental Animals, Capital Medical University (Beijing, China). Mice were kept under standard conditions (temperature, 22 ± 2 °C; humidity, 55 ± 10 %) with a 12:12 light/dark cycle. Food and water were available ad libitum. Animal protocols have been approved by the Animal Care and Use Committee of Capital Medical University.

### Spinal cord injury

Mice were anesthetized with 2% isoflurane. After anesthetization, the T10 spinal cord was exposed by laminectomy, followed by a 70-kilodyne contusion using the Infinite Horizons Impactor (Precision Systems & Instrumentation, Lexington, KY, USA). Afterwards, the muscle and incision opening were sutured. During the surgical procedure and recovery from anesthesia, mice were placed in a warming chamber until they were completely awake. Postoperatively, animals were hydrated with 0.5 ml Ringer’s solution (S. C.) for 5 days. Bladders were voided manually at least twice daily for the duration of the study. Due to the relative ease of bladder expression and lower risk of bladder infections and other complications (e.g., urethral blockages) in female mice compared to males [30], only female mice were used in the current study. Surgical interventions and postoperative animal care were performed in accordance with the guidelines and policies for rodent survival surgery provided by the Experimental Animal Committee of Capital Medical University.

### Experimental groups

Mice were randomly assigned to four groups (Sham, Sham + FMT, SCI, SCI + FMT) with 30 mice in each group. (1) the Sham group underwent a T10 laminectomy without SCI and received vehicle (0.1 ml saline); (2) mice in the Sham + FMT group underwent a T10 laminectomy without SCI and were treated with FMT for 4 weeks; (3) mice in the SCI group were subjected to SCI and were given vehicle; and (4) mice in the SCI + FMT group were subjected to SCI and were treated with FMT for 4 weeks. We gave antibiotics to adult mice (6 weeks of age) in all four groups through drinking water containing 0.2 g/L ampicillin, neomycin, and metronidazole, and 0.1 g/L vancomycin daily for 2 weeks. After the 2-week antibiotics treatment, a total of 100 μl of the resuspended fecal transplant material or vehicle was given by oral gavage to FMT mice (Sham+FMT, SCI + FMT) daily over a period of 4 weeks.

In total, 120 mice were used in this study, with 30 animals in each of the 4 groups. The following were the number of mice allocated for various experiments in each group. Recording of motor-evoked potentials, *n* = 4 in each group; immunohistochemistry on spinal cords and colons, *n* = 4 in each group; Western blotting analysis of spinal cords and colons, *n* = 4 in each group; gastrointestinal motility assay, *n* = 4 in each group; metabolic profiling, *n* = 6 in each group; intestinal permeability assessment by oral gavage of FITC-dextran, *n* = 8 in each group. The same batch of mice used for intestinal permeability assessment (*n* = 8 in each group) were also used for behavioral evaluation (BMS score, BMS subscore, gait analysis and grip strength), and for feces collection for 16S rDNA sequencing and SCFA content determination (two samples of Sham+FMT group were removed from SCFA assay and sequencing due to insufficient sampling).

### Preparation of donor fecal transplant material

The fecal material was collected and isolated as previously reported [31, 32]. The healthy female C57BL/6N mice were kept in the same housing and environmental conditions. Antibiotics-untreated, age-matched healthy female mice were used as donors to collect gut microbiota. The donor’s fecal pellets were collected under SPF conditions. Stools from donor mice were pooled and 100 mg was resuspended in 1 ml of sterile saline (100 mg:1 ml). The solution was vigorously mixed for 10 s using a benchtop vortex (Vortex-Genie 2, Scientific Industries, USA; speed 9), before centrifugation at 800*g* for 3 min. The supernatant was collected and used as transplant material as described below. Donor stool was freshly prepared on the day of transplant within 2 h before gavage administration to prevent changes in bacterial composition.

### Behavioral analysis

The Basso Mouse Scale (BMS) was used to score hindlimb movements as previously described [33]. Animals were assessed in an open field for 4 min before surgery and on days 3, 7, 14, 21, and 28 post-surgery. The performance of left and right hindlimbs was rated separately and averaged to generate BMS scores and subscores. The subscores included analysis of stepping frequency, coordination, paw position, trunk stability, and tail position, which are part of the BMS scoring system [33]. Specific parameters of locomotion were quantified using the DigiGait Image Analysis System [34, 35]. Mice were trained at a speed of 15 cm/s before SCI for 7 days, and then tested at 4 weeks at a speed of 9 cm/s. For each test, at least 5 complete step cycles were recorded, and the movement of each paw was analyzed using the Digigait analysis software (Digigait 12.4). Bilateral hindlimb motor function was evaluated using a grip strength meter [36, 37]. Animals holding a grip bar were rapidly pulled away to assess the peak grip force. Seven consecutive trials were performed weekly for 4 weeks with the highest and lowest values removed to calculate the mean value for each time point.

### Recording of motor-evoked potentials (MEPs)

Animals were anesthetized with pentobarbital sodium (40 mg/kg, i.p.), and two 30-G stimulating electrodes made of stainless steel were placed overlying the left and right motor cortex. MEP was elicited by transcranial electrical stimulation with a pulse of 1 ms at 7 mA using a DS3 constant current isolated stimulator (Digitimer). Responses were recorded from the gastrocnemius muscle using 30-G platinum transcutaneous needle electrodes (distance between recording electrodes was ∼ 1 cm). Recording electrodes were connected to an active headstage (3110 W Headstage; Warner Instruments) and signals amplified using a DP-311 differential amplifier (Warner Instruments). Amplified signal was acquired by the PowerLab 8/30 data-acquisition system (AD Instruments) at a sampling frequency of 20 kHz, digitized, and stored in a computer for analysis.

### Immunohistochemistry

At 4 weeks after SCI, animals were anesthetized with pentobarbital sodium (40 mg/kg, i.p.), and then perfused with 0.1 M PBS (pH 7.4, 37 °C) followed by 4% (w/v) paraformaldehyde in 0.1 M PBS. Frozen sections of the spinal cord and colonic tissues were prepared at 20 μm thickness by using a cryostat microtome (Leica CM 3500, Wetzlar, Germany) and mounted on gelatin-coated glass slides. Sections were equilibrated in 0.1 M Tris-buffered saline for 10 min. After blocking with 10% normal goat serum in PBS for 1 h, sections of spinal cord were incubated for 1 h with primary antibodies, including mouse monoclonal anti-NeuN (1:100, ab177487, Abcam), rabbit polyclonal anti-synapsin (1:100, ab64581, Abcam), or rabbit polyclonal anti-NF-200 (1:100, ab82259, Abcam), and sections of colon were incubated for 1 h with primary antibodies, including rabbit polyclonal anti-ZO-1 (1:100, ab216880, Abcam), or rabbit monoclonal anti-occludin (1:100, ab216327, Abcam). After primary antibody incubation, slides were rinsed in PBS, followed by incubation with secondary antibodies. The slides were coverslipped with glycerinum-mounting media and examined by using a fluorescence microscope. The relative fluorescence intensity was calculated by using Image Pro Plus7.0 (Media Cybernetics, Silver Spring, MD, USA).

Paraffin sections of the spinal cord and colonic tissues were prepared at 8 μm thickness by using a microtome (Leica RM 2235, Wetzlar, Germany) and mounted on gelatin-coated glass slides. Sections were equilibrated in 0.1 M Tris-buffered saline for 10 min. After incubation in 0.3% hydrogen peroxide for 30 min, the sections were permeabilized with 0.1% Triton X-100 for 30 min. After blocking with 10% normal goat serum in PBS for 1 h, sections of spinal cord were incubated for 1 h with primary antibodies, including rabbit polyclonal anti-TNFα (1:100, ab6671, Abcam), rabbit polyclonal anti-IL-1β (1:100, ab9722, Abcam), or rabbit polyclonal anti-NF-κB (1:100, ab16502, Abcam). After primary antibody incubation, slides were rinsed in PBS, followed by incubation with secondary antibodies. The slides were coverslipped with glycerinum-mounting media and examined by fluorescence microscopy. The relative gray value was calculated by using Image Pro Plus7.0 (Media Cybernetics, Silver Spring, MD, USA).

### Metabolic parameters

Metabolic parameters were measured by using metabolic phenotyping chambers (Mouse Promethion Continuous caging system; Sable Systems, Las Vegas, NV). To start the metabolic phenotyping, animals were transferred to the chambers individually under normal housing conditions. Air within the cages was sampled through micro-perforated stainless steel sampling tubes located around the bottom of the cages, above the bedding. Gas sensors were calibrated before each run with 100% N_2_ as reference value zero. The incurrent flow rate was set at 2000 mL/min and gases were sampled continuously for each cage, from multiple points within the cage (250 ml/min). Oxygen consumption and carbon dioxide (CO_2_) production were recorded for each mouse. Respiratory exchange quotient (RQ) was calculated as the ratio of CO_2_ production over O_2_ consumption. Energy expenditure was calculated using the Weir equation: Kcal/h = 60 × (0.003941 × VO_2_ + 0.001106 × VCO_2_) [38]. Values were calculated after application of algorithms using macros provided with the analysis software ExpeData [39].

### FITC-dextran permeability assay

Four weeks after injury, the mice were fasted for 14 h and gavaged with fluorescein isothiocyanate-dextran (FITC-dextran, 4 KD; Sigma-Aldrich, Madrid, Spain) at a dose of 60 mg per 100 g body weight in a volume of 0.2 ml. Four hours later, the blood were collected by cardiac puncture and clotted for 30 min, followed by 90 s centrifugation at 6000*g*. Serum was equally diluted with PBS, and 100 μl dilution was measured in a 96-well plate using a plate reader (EnSpire; Perkin Elmer) at an excitation of 481 nm and an emission of 524 nm. The FITC-dextran was quantified by referring to standard curve measurements in the same plate.

### Gastrointestinal transit assessment

GI motility was assessed by using radiographic methods as described previously at 4 weeks following injury [40]. In detail, animals were intragastrically gavaged of barium (0.2 ml, 2 g/ml) and immobilized in a prone position by using an adjustable hand-made transparent plastic tube. The plain facial radiographs of the GI tract were obtained using a digital X-ray apparatus (Siemens; 50 kV, 10 mA) and processed with NPG Real DVD Studio II software. Exposure time was adjusted to 0.06 s. To further reduce stress, mice were released immediately after each image shot (immobilization lasted 1-2 min). X-rays were recorded at different times (immediately and 0.5, 1, 2, 3, 4, 6, and 8 h after administration of barium). Analysis of the radiographs was performed by a trained investigator blind to the different groups. Alterations in GI motility were semiquantitatively determined from the images by assigning a compounded value to indicated regions of the GI tract considering the following parameters: percentage of the GI region filled with contrast (0-4); intensity of contrast (0-4); homogeneity of contrast (0-2); and sharpness of the GI region profile (0-2). Each of these parameters was scored and a sum (0–12 points) was made. X-ray images were also morphometrically analyzed using ImageJ software (version 1.38, National Institute of Health, USA), and the alterations in the stomach and colorectum were quantified.

### Microbial DNA extraction and PCR amplification

Microbial DNA was extracted from stool samples using the E.Z.N.A.® Stool DNA Kit (Omega Bio-tek, Norcross, GA, U.S.) according to the manufacturer’s protocols. The V3-V4 region of the bacteria 16S rDNA gene was amplified by using PCR (95 °C for 2 min, followed by 25 cycles at 95 °C for 30 s, 55 °C for 30 s, and 72 °C for 30 s and a final extension at 72 °C for 5 min) using primers 338F 5′- ACTCCTACGGGAGGCAGCA-3′ and 806R 5′- GGACTACHVGGGTWTCTAAT-3′. PCR reactions were performed in triplicate 20 μL mixture containing 4 μL of 5 × FastPfu Buffer, 2 μL of 2.5 mM dNTPs, 0.8 μL of each primer (5 μM), 0.4 μL of FastPfu Polymerase, and 10 ng of template DNA.

### Illumina MiSeq sequencing

Amplicons were extracted from 2% agarose gels, purified by using the AxyPrep DNA Gel Extraction Kit (Axygen Biosciences, Union City, CA, USA), and quantified by using QuantiFluor™ -ST (Promega, USA). Purified amplicons were pooled in equimolar and paired-end sequenced (2 × 300 bp) on an Illumina MiSeq platform according to the standard protocols.

### Processing of sequencing data

Raw fastq files were quality-filtered by Trimmomatic and merged by FLASH with the following criteria: (i) The reads were truncated at any site receiving an average quality score < 20 over a 50-bp sliding window. (ii) Sequences whose overlap being longer than 10 bp were merged according to their overlap with mismatch no more than 2 bp. (iii) Sequences of each sample were separated according to barcodes (exact match) and primers (allowing 2 nucleotide mismatch), and reads containing ambiguous bases were removed.

### Fecal SCFA detection

Fecal samples were weighed and then 20 mg transferred into a 2 ml EP tube, and 1 mL phosphoric acid (0.5% v/v) solution was added in the EP tube, followed by vortexing for 10 min, and ultrasonic wave treatment for 5 min. Then 0.1 mL supernatant was added to a 1.5 mL centrifugal tube, and 0.5 mL MTBE (containing internal standard) solution was added, followed by vortexing for 3 min, and ultrasound treatment for 5 min. After that, the samples were centrifuged for 10 min at 12,000 r/min at 4 °C. After centrifugation, the supernatant was analyzed using a gas chromatography-mass spectrometry (GC-MS/MS 7890B-7000D; Agilent Technologies Inc.) on a silica capillary column (DB-FFAP, 30 m × 0.25 mm × 0.25 μm, Agilent J&W) under the following conditions: injected sample size, 2 μL, splitless; injector temperature at 200 °C; initial oven temperature at 95 °C, held for 1 min, raised to 100 °C at a rate of 25 °C/min, raised to 130 °C at a rate of 17 °C/min, held for 0.4 min, raised to 200 °C at a rate of 25 °C/min, and held for 0.5 min. Helium was used as a carrier gas at 1.2 ml/min.

The main mass spectrometry conditions were as follows: ion source, EI; transfer line temperature, 230 °C; ion source temperature, 230 °C; quad temperature, 150 °C, electron energy, 70 eV; scan mode, MRM; and solvent delay, 2.4 min.

### Western blot analysis

A section of spinal cord 1 cm in length (at T9 ~ T11, with epicenter as the center, extending 5 mm to the cranial end and caudal end, respectively) and colonic tissue 1 cm in length (1 cm to cecocolic junction) were collected. The total protein was prepared in a lysis buffer (Beyotime, China) by lysing tissue homogenates for 1 h, and then centrifuged at 14,000*g* for 8 min at 4 °C. The protein content of the supernatant was determined by using a protein assay kit (BCA, Pierce, Rockford, IN, USA). Equal amounts of total protein (50 μg) were separated by using 12% sodium dodecyl sulfate-polyacrylamide gel electrophoresis and transferred to polyvinylidene difluoride membranes. The membranes were blocked with 5% non-fat skim milk in Tris-buffered saline solution with 0.05% Tween-20 (TBST) for 1 h, and then incubated with antibodies against TNFα (1:100, ab6671, Abcam), IL-1β (1:100, ab9722, Abcam), or NF-κB (1:100, ab16502, Abcam) at 4 °C overnight. After washing 3 times with TBST, appropriate horseradish peroxidase-conjugated secondary antibodies were added. β-actin (1:1000, Cell Signaling Technology) was used as an internal control. The bands were visualized by using enhanced chemiluminescence, and images were acquired with ChemiDoc MP System (Bio-Rad, Hercules, CA, USA). The relative band intensities were quantified by using Quantity One (Bio-Rad, Hercules, CA, USA).

### Bioinformatics and statistical analysis

Demultiplexed raw reads were quality-filtered and cleaned from chimeric reads, and the feature table was generated using the dada2 denoise-paired option in the Qiime 2 plugin [41, 42]. The dada2 plugin was used for sequence quality control. Sequences were clustered into amplicon sequence variants (ASVs) which could be thought of as 100% operational taxonomic units (OTUs). The naive Bayes classifier and the q2-feature classifier plugin with the reference database (SILVA Release 138) were used to assign taxonomy to the sequences and map the sequences. Determination of alpha and beta diversities and analysis of similarity (ANOSIM) were also conducted in Qiime 2. The ace and chao alpha-diversity indices were calculated on the data at the ASV level. The beta-diversity analysis was performed via principle coordinate analysis (PCoA) on Bray-Curtis dissimilarity matrix.

For taxa among groups, Kruskal-Wallis H-test was applied (Tukey–Kramer post hoc tests were further examined) [43]. Moreover, we applied analysis of composition of microbiomes (ANCOM) to analyze each individual taxa abundance among groups [44]. The ANCOM was developed for analyzing 16S rDNA gene sequencing data, which makes no distributional assumptions, allows for adjustment of covariates, and was reported to reduce false positive rate while increase statistical power. ANCOM determines the final significance of a taxon by using the empirical distribution of W. The significance of each test is determined using the Benjamini-Hochberg procedure that controls for false discovery rate (FDR) at 0.05. Results were presented as the mean values with the standard error of mean (SEM). The data were analyzed by using SPSS, version 17.0 statistic software package (SPSS Inc., Chicago, Illinois, USA). Student’s *t* tests were used to determine significance between two groups. Multivariate analysis of variance (MANOVA) was conducted to test between-group differences on the dependent measures. One-way analysis of variance followed by post hoc Tukey’s analysis was performed to compare groups of three or more. In addition, relationships between significant SCFA changes and behavior scores (BMS), BMS subscores and gut permeability were evaluated using the Pearson correlation method. All analyses were conducted with an alpha level of *p* < 0.05 using SPSS statistical software. Effect size was reported as either a *R*^2^ for the correlation analysis or Partial Eta2 for MANOVA.

## Results

### FMT treatment improves locomotor recovery in spinal cord injury mice

**Fig. 1:**
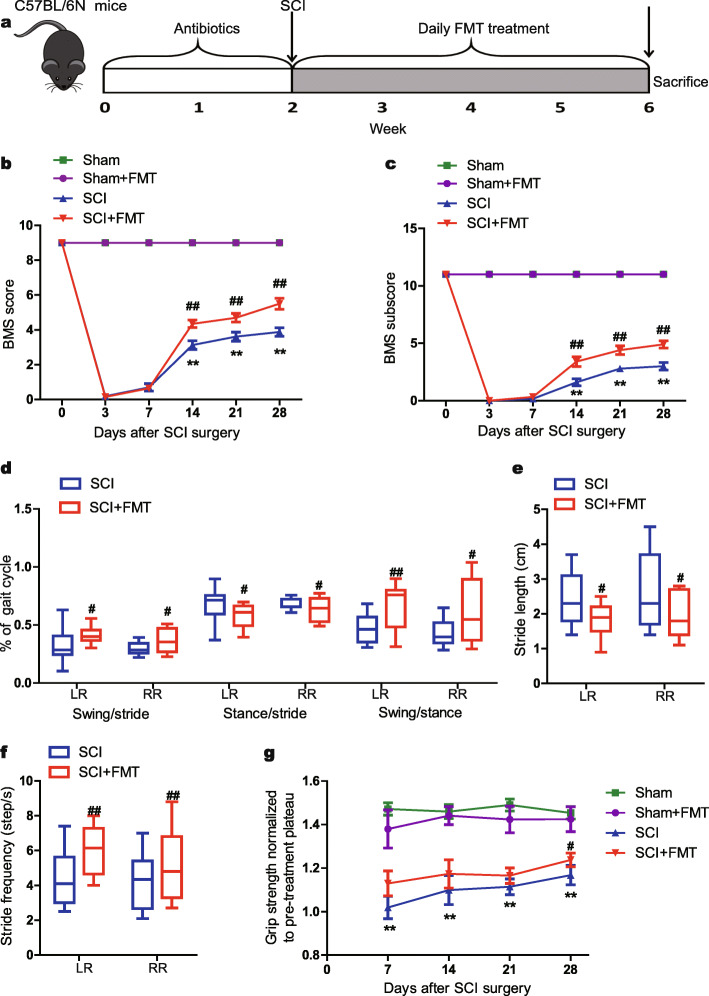
Effect of FMT treatment on locomotor recovery. **a** Schematic representation of the experiments. **b, c** Time course of locomotor functional recovery as assessed by BMS (**b**) and BMS subscore (**c**). **d–f** Gait analysis by using an automated treadmill (DigiGait). **d** Swing to stride ratio, stance to stride ratio, and swing to stance ratio. **e** Stride length and **f** stride frequency. **g** Hindlimb grip strength. Data were normalized to pretreatment (post-injury) baseline with a value “1” referring to no difference after treatment and values above “1” indicating improvement. *p < 0.05 compared to Sham group; **p < 0.01 compared to Sham group; #*p* < 0.05 compared to SCI group; ##*p* < 0.01 compared to SCI group. LR, left rear; RR: right rear

To investigate the effects of gut microbiota on SCI, we conducted FMT from healthy uninjured mice to mice that had been subjected to spinal cord injury (Fig. 1a). After microbiota depletion with antibiotics, mice were randomly assigned to four groups (Sham, Sham + FMT, SCI, SCI + FMT), FMT mice (Sham+FMT, SCI + FMT) received stool material from healthy mice, and other groups received vehicle. All the mice were housed and sacrificed at the end of week 6. The locomotor recovery was observed during the 4 weeks post-injury in SCI groups. FMT treatment further significantly increased hindlimb locomotor function starting from 14 days after injury compared to that of SCI group, and the improvement in BMS scores and BMS subscores continued until the end of the experiment (Fig. 1b, c). Moreover, digigait and grip analysis was complementary behavioral tests used to evaluate the hindlimb gait and strength. The percentage of stride duration spent in the swing phase was decreased in SCI mice and this parameter was significantly improved in FMT treatment group (Fig. 1d). The percentage of the stride duration spent in the stance phase was significantly reduced following FMT treatment compared to SCI group (Fig. 1d). Accordingly, FMT treatment resulted in a significant increased swing to stance ratio in SCI mice (Fig. 1d). In addition, stride length was increased and stride frequency was decreased, which was reversed by FMT treatment (Fig. 1e, f). To a certain extent, FMT was able to ameliorate the gait abnormality induced by SCI and increase the coordination of gait. Measurements of grip strength were presented as a percentage of the baseline grip strength. The SCI mice demonstrated a gradual continued recovery of grip strength after injury. After treatment, grip strength differed between SCI group and FMT group showing a nonsignificant trend of improvement at 7 day post-injury (dpi), 14 dpi, and 21 dpi. Only at 28 dpi, the FMT treatment group demonstrated a statistically significant improvement compared to the SCI group (Fig. 1g). Altogether, mice treated with FMT exhibited a relatively greater locomotor recovery compared to injured mice without FMT treatment.

### FMT treatment facilitates restoration of descending motor pathways

**Fig. 2:**
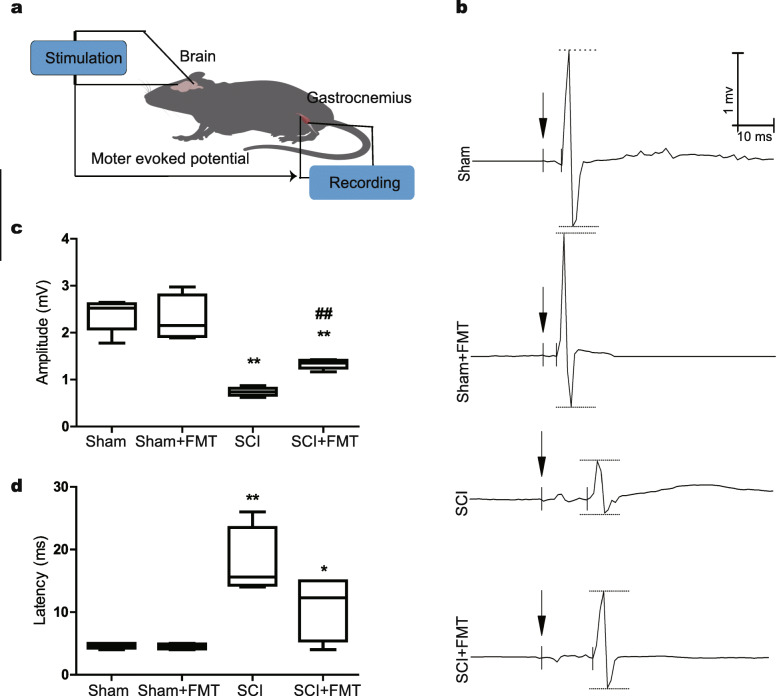
Effect of FMT treatment on spinal cord conduction capability. **a** A schematic diagram of MEP recording experiment. MEP was recorded from the gastrocnemius muscle in an anesthetized state after electrical stimulation on the motor cortex. **b** Representative MEPs recorded from Sham mice and SCI mice that had received the indicated treatment 4 weeks after injury. The arrows represent stimulation. **c, d** The amplitude (**c**) and the latency (**d**) were quantified and statistically analyzed. *p < 0.05 compared to Sham group; **p < 0.01 compared to Sham group; ##*p* < 0.01 compared to SCI group

Next, to examine the function of descending pathways from the motor cortex to the hindlimb motor neurons, we recorded motor-evoked potentials (MEPs) at 4 weeks after injury (Fig. 2). MEPs, which reflect the connectivity of neuromuscular unit, were recorded at the gastrocnemius muscle after electrical stimulation on the motor cortex (Fig. 2a). In Sham and Sham+FMT groups, typical waveforms of MEPs were detected. Following injury, the MEPs were mostly abolished, indicating a disruption of the neuromuscular unit. With FMT treatment, the SCI mice displayed a larger amplitude, compared to that of SCI mice without FMT treatment (Fig. 2b). The quantification of the amplitudes in different groups is shown in Fig. 2c. Additionally, SCI mice developed a significantly greater MEP latency (to 17.8 ms) compared with that of Sham group (4.63 ms). The latencies of MEP showed no significant difference between SCI and SCI + FMT group (Fig. 2d). These observations indicated that FMT treatment is beneficial and may further enhance functional recovery after SCI.

### FMT treatment promotes neuronal survival and synaptic regeneration

**Fig. 3:**
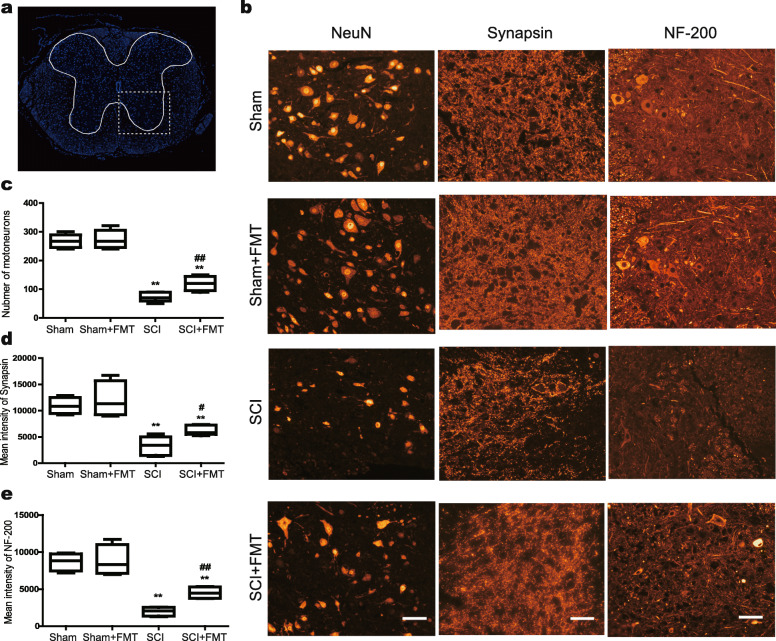
Effect of FMT treatment on neuronal survival and synaptic regeneration following SCI. **a** DAPI staining of spinal cord section with outlined gray matter area and a squared inset of observed ventral horn. **b** Immunostaining of NeuN, Synapsin, and NF-200 in the T10 region of spinal cords in different groups. **c–e** Quantification of NeuN-positive neuronal cell bodies (**c**), synapsin immunoreactivity (**d**), and NF-200 immunoreactivity (**e**) of the ventral horn. Scale bar, 50 μm. *p < 0.05 compared to Sham group; **p < 0.01 compared to Sham group; #*p* < 0.05 compared to SCI group; ##*p* < 0.01 compared to SCI group

To investigate the anatomical basis of the observed locomotor recovery, we performed immunofluorescent staining on the spinal cord sections (Fig. 3). In the Sham group, neurons in the ventral horn appeared normal, with large cell bodies and intact axons. SCI induced a significant loss of NeuN-positive neurons. However, the number of NeuN-positive cells was significantly higher in the FMT group compared to that of the SCI group (Fig. 3a, b). The quantification of neuronal cell bodies across the four groups is shown in Fig. 3c. Synapsin (SYN) is a type of vesicle protein that marks the presynaptic membranes. Immunostaining analysis showed a reduction in synapsin staining in the SCI group at 4 weeks post-injury, and the staining intensity was increased by FMT treatment (Fig. 3b, d). Neurofilaments (NF-200) are cell type-specific proteins abundant in neuronal axons, and the staining has been applied for evaluation of neuronal and axonal damage [45, 46]. Compared with the SCI group, staining of NF-200 in the lesion areas was increased with FMT treatment at 4 weeks post-injury (Fig. 3b, e). The data suggested that FMT treatment may have promoted neuronal survival and axonal regeneration after traumatic SCI.

### FMT treatment improves weight gain and metabolic profile in SCI mice

**Fig. 4:**
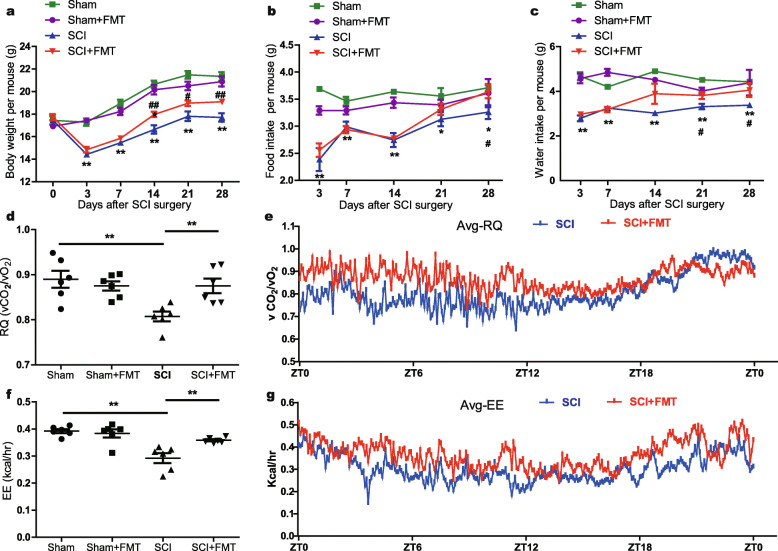
Effect of FMT treatment on body weight, food intake, water consumption, and metabolism. **a** Changes in body weight over time in Sham, Sham+FMT, SCI, and SCI + FMT groups. **b**, **c** food intake (**b**) and water consumption (**c**) were examined during the 4 weeks in the four groups. **d–g** Respiratory quotient (RQ) (**d**) and energy expenditure (EE) (**f**) were measured at the end of the experiments. The mean respiratory quotient (Avg_RQ) (**e**) and mean energy expenditure (Avg_EE) (**g**) were measured every 5 min for 24 h in SCI group and SCI + FMT group. *p < 0.05 compared to Sham group; **p < 0.01 compared to Sham group; #*p* < 0.05 compared to SCI group; ##*p* < 0.01 compared to SCI group

The body weights were monitored and compared at indicated time points. The Sham and Sham+FMT groups showed stable and comparable body weights over the 4 weeks of observation. For both SCI and SCI + FMT groups, the body weights rapidly decreased during the first 3 days after surgery, and gradually gained back in the following days. Compared with SCI group, SCI + FMT mice gained more weight, which showed statistically significant difference at 14, 21, and 28 days post-injury (Fig. 4a). Consistent with these results, an increase in food intake and water consumption was observed in the SCI + FMT group as compared to the SCI group, especially at day 28 post-injury (Fig. 4b, c). In addition, the metabolic parameters were assessed over a 24-h period at week 4 post-injury. Indirect calorimetry revealed that the average respiratory exchange quotient (RQ) values were restored in SCI + FMT group (Fig. 4d, e). As shown in Fig. 4f, g, the average energy expenditure was also significantly elevated with FMT treatment in SCI animals. These data demonstrated that FMT treatment after SCI led to more food and water consumption, higher energy expenditure, and greater body weight gains.

### FMT treatment is conducive to maintaining intestinal barrier integrity in SCI mice

**Fig. 5:**
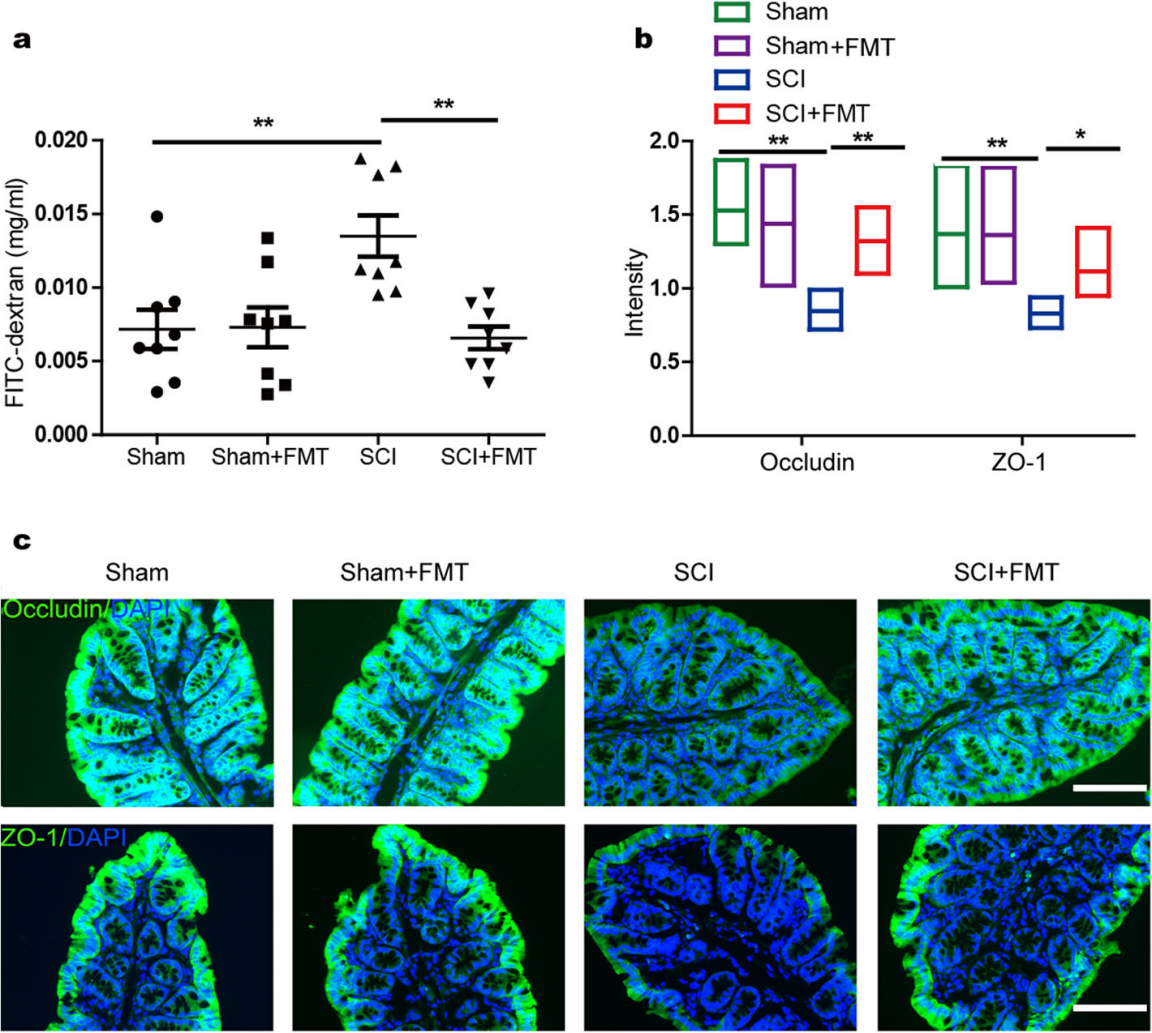
Effect of FMT treatment on intestinal permeability and expression of tight junction proteins. **a** Intestinal permeability was assessed 4 weeks following injury by measuring FITC intensity in serum after oral gavage of FITC-dextran. **b, c** Quantification of occludin immunoreactivity and ZO-1 immunoreactivity (green) with representative immunofluorescence images of colon sections. DAPI, blue; *p < 0.05 compared to SCI group; **p < 0.01 compared to SCI group; Scale bar, 50 μm

Altered intestinal barrier integrity and subsequent gastrointestinal dysfunction have been postulated as a pathophysiological event in SCI. To test whether FMT treatment had any impact on intestinal barrier permeability after SCI, the mice were gavaged with FITC-labeled dextran (4 KD) at week 4 post-injury, and FITC levels in blood were measured. Permeability in colon was more severe in SCI mice compared to that of SCI + FMT mice (Fig. 5a). Intestinal tight junctions (TJs) have been shown to be associated with intestinal barrier integrity [47]. Therefore, we investigated the expression and distribution of TJ proteins in the colon. As shown in Fig. 5b, c, expressions of ZO-1 and occludin indicated an increased disruption and disorganization at the apical surface, and FMT treatment stabilized TJ structures, as evidenced by smooth and organized localization of ZO-1 and occludin (Fig. 5b). The results demonstrated that FMT treatment had led to an upregulation of tight junction protein expression and facilitated to maintain intestinal barrier integrity after SCI.

### FMT treatment accelerates GI transit in SCI mice

**Fig. 6:**
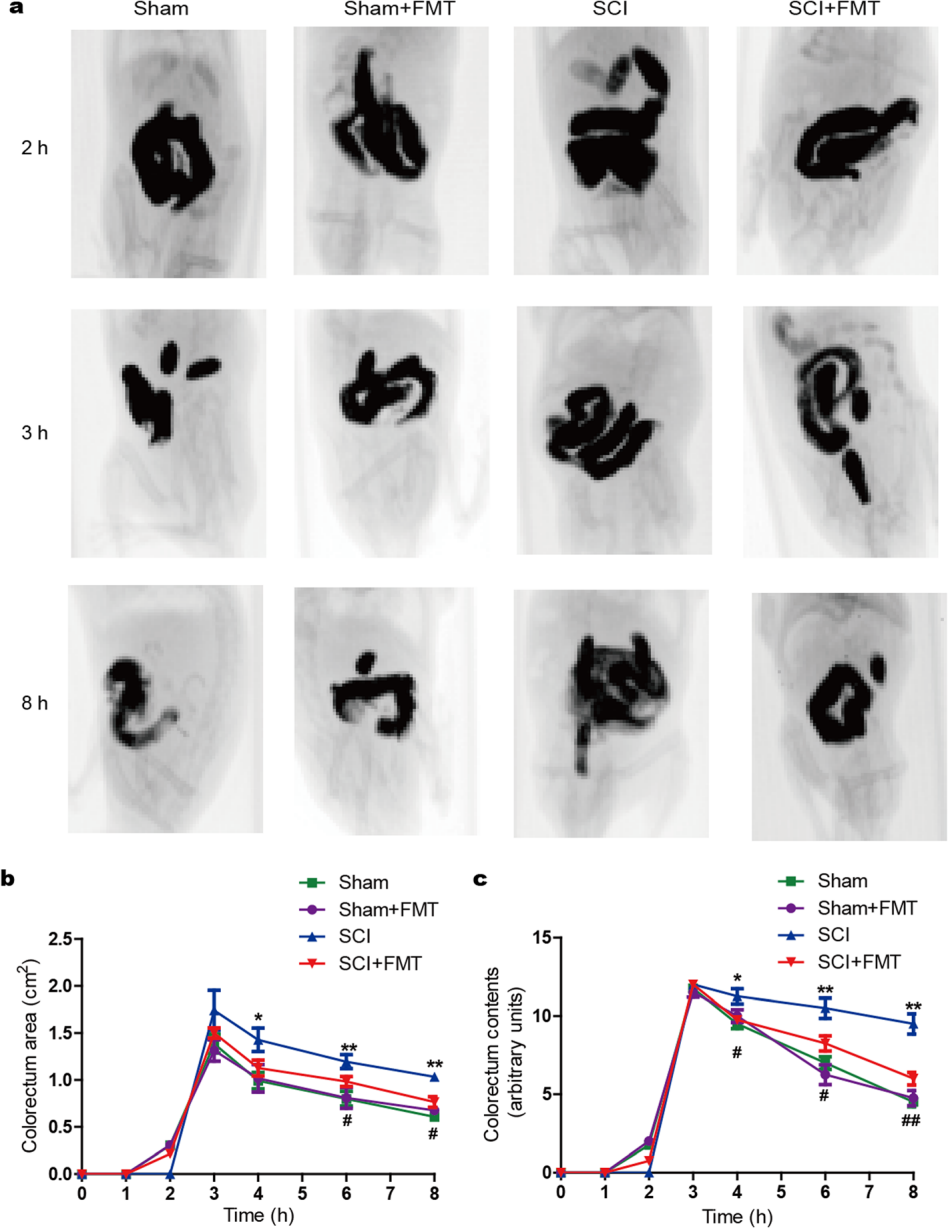
Effect of FMT on gastrointestinal motility in SCI mice. **a** Representative images of Sham, Sham+FMT, SCI, and SCI + FMT groups at 2, 3, and 8 h after administration of barium. **b** Filling of colorectum was measured by radiological methods. **c** Colorectum size was determined by using ImageJ. **p < 0.01 compared to Sham group; #*p* < 0.05 compared to SCI group; ##*p* < 0.01 compared to SCI group

GI transit, as an overall measure of GI motility, was assessed in mice by using barium gavage followed by X-ray imaging. Representative images from different groups are shown in Fig. 6a at different time points (2, 3, and 8 h). Overall, SCI mice displayed a higher level of filling of GI tract, indicative of a slower GI transit, while FMT treatment accelerated GI transit in mice after injury (Fig. 6a). With regard to filling of colorectum, the dynamics for the Sham+FMT group was similar to that for the Sham group. GI motility was markedly delayed after injury, which was improved at certain time points in SCI + FMT mice (Fig. 6a, b). Morphometric analysis showed that injury increased the filling of colorectum, which was reversed by FMT treatment (Fig. 6a, c). Collectively, FMT treatment might have contributed to the faster GI transit following SCI.

### FMT treatment modulates gut microbiota composition in SCI mice

**Fig. 7:**
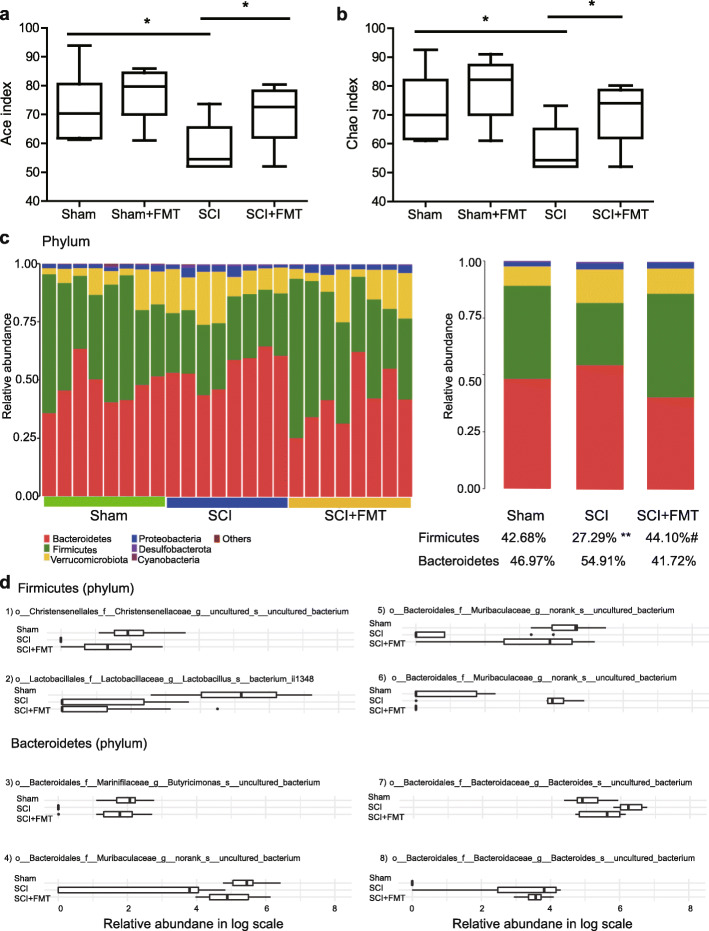
Effect of FMT treatment on gut bacterial composition after SCI. **a, b** Comparison of ace index (**a**) and chao index (**b)** based on OUT levels in the four groups. **c** Bacterial composition of the different communities at the phylum level and quantitative analyses of relative abundances of *Firmicutes* and *Bacteroidetes* among different groups. **d** Differentially relative abundance of taxa in log scale among Sham, SCI, and SCI + FMT groups. All taxonomic entities appeared as differentially abundant at FDR = 0.05. (Feature ID: 1) 2fbb5c1c08b57ab1779d169c4ab3eba0; 2) 1dbb5d4c766a2a485d9bb3ac14152d2a; 3) dadce561323d0f5dab4fe19071e1d518; 4) ba29819fff46ad80af70cb2ee620b6c9; 5) fe94fedbf48562eea97a74ec33a58fab; 6) 1aa1ab2ef3c4a9dadb258dd983a5b1d4; 7) 00d0f4d040a89105ce2610357ba60947; 8) 642062eb68894277cebbf080abdda26b). *p < 0.05 compared to Sham group; **p < 0.01 compared to Sham group; #*p* < 0.05 compared to SCI group

To test whether FMT had modulated gut microbiota, we performed 16S rDNA (V3 + V4 regions) gene sequencing to analyze the bacterial taxonomic composition following microbial therapy in SCI mice. As shown in Fig. 7a, b, significant differences existed in the ace and chao indices between the Sham group and SCI group, suggesting that SCI had a marked impact on the richness of microbiota during the recolonization process following antibiotics treatment, and this observation was in agreement with our previously published clinical data as to SCI patients vs. healthy controls [21]. FMT treatment significantly increased the richness of the intestinal microbiota in SCI mice. To measure the degree of similarity between microbial communities, β diversity was further evaluated by using Bray-Curtis principal coordinate analysis (PCoA) (Fig. S1). The Sham+FMT group clustered closely to the Sham group, suggesting that antibiotics-treated Sham mice (without SCI) may undergo recolonization of gut microbiota, and FMT treatment did not significantly impact the recolonization state 4 weeks after antibiotics administration had ceased. Nevertheless, it is possible that FMT might have affected the dynamics of the recolonization process, particularly at an earlier time point, which requires further study. ANOSIM of beta-diversity analysis revealed significant differences in the structure of the gut microbiota among Sham, SCI and SCI + FMT groups at the ASV level (Fig. S2), phylum level (Fig. S3), and genus level (Fig. S4). The differences in microbial communities among groups suggested that gut dysbiosis in SCI mice was modulated following FMT treatment. By investigating the abundance and distribution of gut microbiota in the Sham group, SCI group, and SCI + FMT group, we identified a number of altered microbiota that might be responsible for microbial dysbiosis. The heatmap showed a significant difference in the relative abundance across the groups at the phylum level (Fig. 7c). Compared to the Sham group, the relative abundance of *Firmicutes* was reduced in the SCI group, which was reversed by FMT treatment. In contrast, the relative abundance of *Bacteroidetes* in SCI mice did not differ from those of Sham and SCI + FMT mice (Fig. 7c). To further explore changes in gut microbial community between groups, analysis at the genus level was performed. As shown in Fig. S5, SCI significantly decreased the relative abundance of *Blautia*, *Anaerostipes*, and *Lachnospiraceae_NK4A136*, and significantly increased the relative abundance of *Bilophila*. FMT treatment markedly increased the relative abundance of *Blautia*, *Anaerostipes*, and *Lachnospiraceae_NK4A136* and decreased that of *Bilophila*. We also analyzed the data using ANCOM which accounts for the underlying structure in the data and can be used for comparing the composition of microbiomes in two or more populations. Analyses for each taxon using ANCOM models revealed that SCI significantly decreased the abundance of *Christensenellaceae* and *Lactobacillus* belonging to phylum *Firmicutes*. FMT treatment markedly increased the abundance of *Christensenellaceae*, while it had little effect on that of Lactobacillus (Fig. 7d). Also, the abundances of *Butyricimonas*, *Muribaculaceae*, and *Bacteroides* belonging to phylum *Bacteroidetes* were significantly altered between Sham group and SCI group. FMT treatment significantly increased the abundance of *Butyricimonas* in SCI mice (Fig. 7d). A supplemental table with statistics between the groups is shown in Table S2. Together, these data indicated that FMT treatment could have modulated the microbiota composition to alleviate gut microbial dysbiosis in SCI mice.

### FMT treatment restores fecal short-chain fatty acids (SCFAs) in SCI mice

**Fig. 8:**
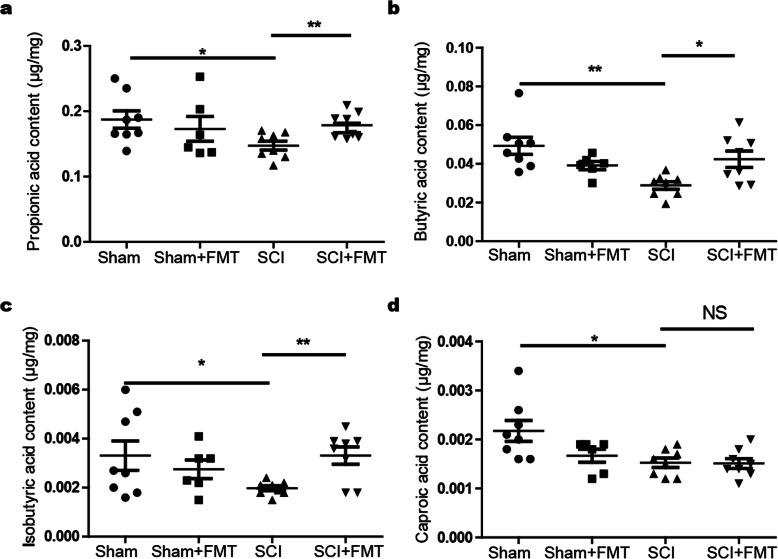
Effect of FMT treatment on fecal SCFA levels in mice. The SCI-mediated decrease of propionic acid, butyric acid, and isobutyric acid expression was ameliorated by FMT treatment. **a** Fecal propionic acid content. **b** Fecal butyric acid content. **c** Fecal isobutyric acid content. The SCI-mediated decrease of caproic acid expression was not significantly changed by FMT treatment. **d** Fecal caproic acid content. SCFAs were analyzed by GC-MS. *p < 0.05 compared to SCI group; **p < 0.01 compared to SCI group; NS, not significant

Certain end products of fermentation by the gut microbes could enter the bloodstream and impact the physiology of the CNS in the host [6, 48]. Among the potential factors regulating the microbiota gut-brain axis, microbial metabolites, SCFAs, may be the major mediators. We examined the fecal concentrations of certain SCFAs including acetic acid, propionic acid, butyric acid, isobutyric acid, valeric acid, isovaleric acid, and caproic acid in the different groups. Among the tested fecal SCFAs, butyric acid content was altered to the greatest extent, a decrease by 58.6% in SCI compared to that of the Sham group. FMT upregulated fecal butyric acid level by 46.7% compared to that of SCI mice (Fig. 8b). Similar to butyric acid, propionic acid and isobutyric acid were also decreased in SCI mice by 21.2% and 39.4%, respectively, compared to those of the Sham group, and FMT treatment significantly augmented fecal propionic acid and isobutyric acid levels by 21.3% and 65.0% in SCI mice, respectively (Fig. 8a, c). Caproic acid was decreased by 31.8% in SCI mice, but FMT treatment did not significantly change the amount of caproic acid (Fig. 8d). Additionally, no significant changes were found as to acetic acid, valeric acid, and isovaleric acid (Table S1). The data suggested that FMT treatment might have mediated pathophysiological changes in the host through alteration of fecal SCFAs.

### Correlations between SCFAs and locomotor recovery/intestinal barrier permeability

We next assessed whether the contents of propionic acid, butyric acid, and isobutyric acid were correlated with locomotor recovery or intestinal integrity. A multivariate analysis of variance (MANOVA) was performed with groups as the independent variable, motor outcomes (BMS scores and subscores), intestinal permeability (FITC-dextran), and level of metabolites (butyric acid, propionic acid, and isobutyric acid) as the dependent variables. Hotelling’s trace revealed a significant multivariate effect of group on corresponding index [*F*(12,30) = 33.83; *p* < 0.001]. Univariate ANOVAs revealed significant effects among different groups on BMS scores [*F*(2,21) = 97.04; *p* < 0.001], BMS subscore [*F*(2,21) = 210.79; *p* < 0.001], FITC-dextran [*F*(2,21) = 10.04; *p* = 0.001], butyric acid [*F*(2,21) = 7.79; *p* = 0.003], propionic acid [*F*(2,21) = 4.76; *p* = 0.02], and isobutyric acid [*F*(2,21) = 3.61; *p* = 0.045].

**Fig. 9:**
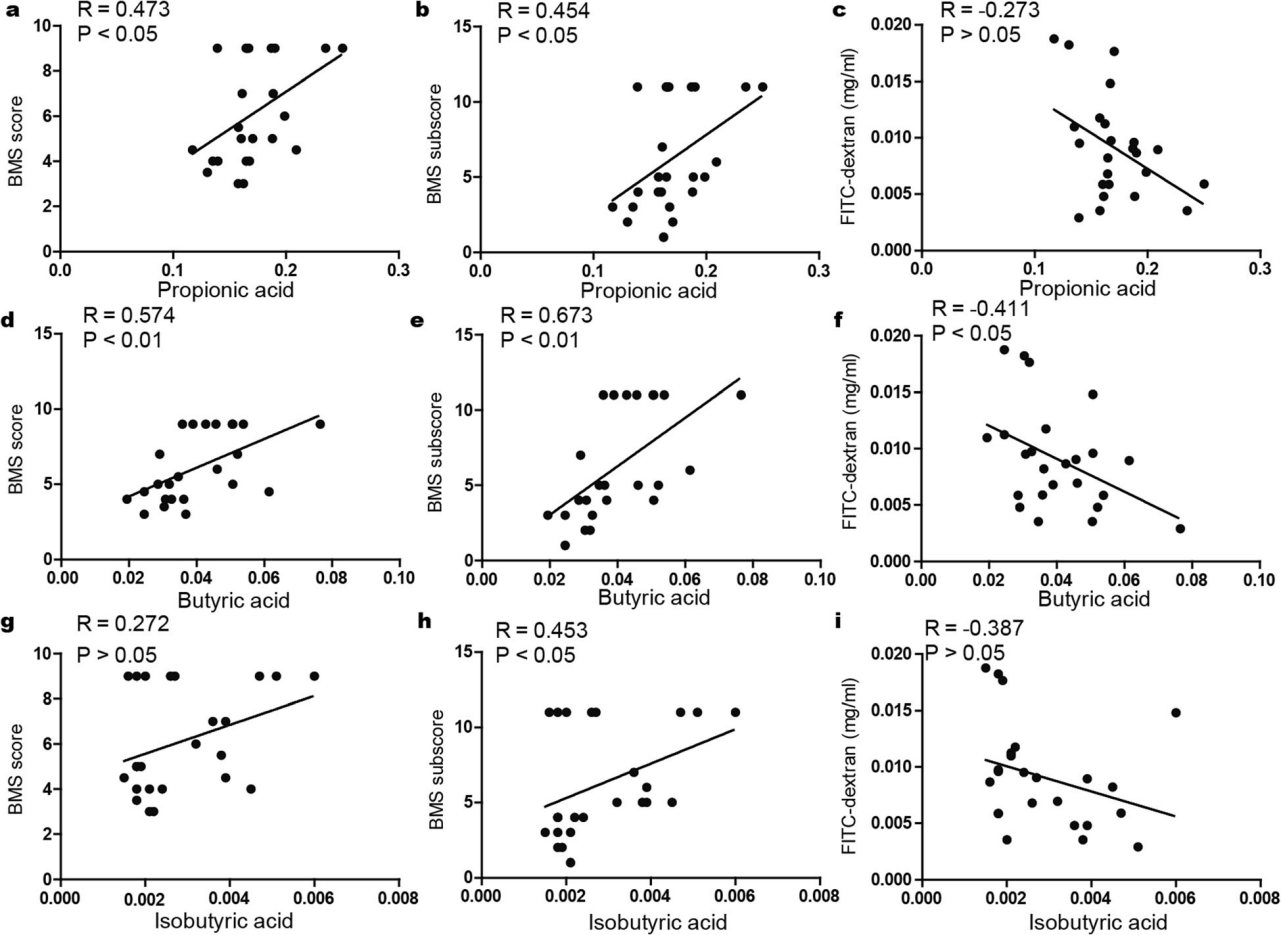
Correlations between SCFAs and BMS scores/BMS subscores/FITC-dextran permeability. **a–c** Correlations between propionic acid and BMS scores (**a**), BMS subscores (**b**), and FITC-dextran permeability (**c**). **d–f** Correlations between butyric acid and BMS scores (**d**), BMS subscores (**e**), and FITC-dextran permeability (**f**). **h–g** Correlations between isobutyric acid and BMS scores (**h**), BMS subscores (**i**), and FITC-dextran permeability (**g**)

As shown in Fig. 9, the content of propionic acid was positively correlated with open-field locomotor (BMS) scores and BMS subscores; similar results were obtained with regard to the correlation of butyric acid amount to BMS scores and BMS subscores. Inverse relationships were found between the content of butyric acid and FITC-dextran permeability. In addition, the content of isobutyric acid was positively correlated with BMS subscores. The association between the content of isobutyric acid and BMS scores / FITC-dextran permeability was not statistically significant. It seems that the contents of certain SCFAs could provide a degree of predictive power as to functional recovery and barrier integrity after SCI.

### FMT alleviates neuroinflammation possibly by suppressing IL-1β/ NF-κB signaling pathway

**Fig. 10:**
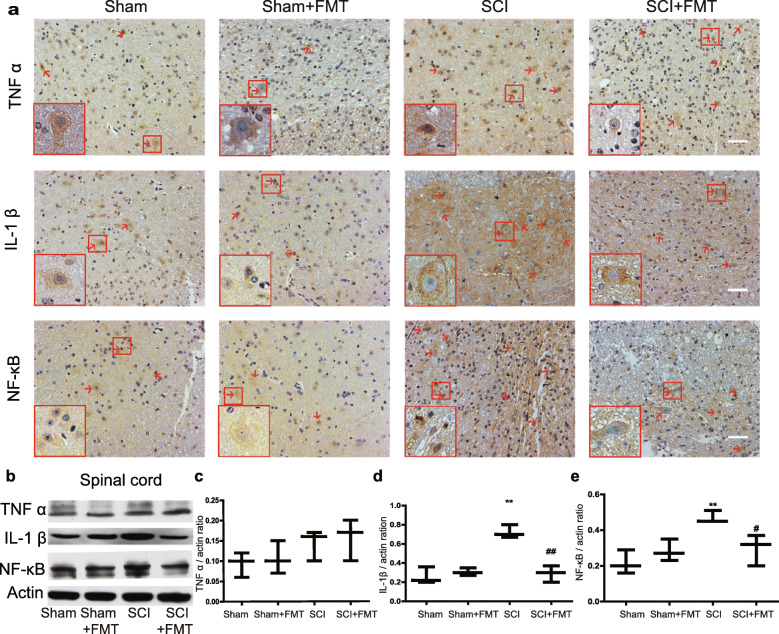
Effect of FMT treatment on expression of TNF α, IL-1β, and NF-κB in spinal cord. **a** TNFα, IL-1β, and NF-κB were stained by immunohistochemistry on spinal cord sections from each group. Scale bar, 50 μm. **b** The expression of TNF α, IL-1β, and NF-κB was detected by western blot, and the relative amounts of TNFα (**c**), IL-1β (**d**), and NF-κB (**e**) were semi-quantified. (Red arrows point to the positive staining. Insets have been embedded to show higher magnification.) **p < 0.01 compared to Sham group; #*p* < 0.05 compared to SCI group; ##*p* < 0.01 compared to SCI group

To further explore the molecular interactions between gut microbial dysbiosis and neuroinflammation in SCI, we characterized spinal cord expression of TNFα, IL-1β, and NF-κB by applying immunohistochemical analysis and western blotting (Fig. 10a, b). Figure 10c indicates that the expression of TNFα did not show significant alterations among different groups. Meanwhile, a substantial increase in IL-1β- and NF-κB-positive staining was found in spinal cord tissues collected from mice at 4 weeks after SCI. Furthermore, treatment with FMT reduced the positive staining of both IL-1β and NF-κB (Fig. 10a). The result of western blotting indicated that FMT treatment remarkably attenuated the expressions of IL-1β and NF-κB following injury (Fig. 10d, e) and did not significantly alter the expression of TNFα (Fig. 10c), which was consistent with the immunostaining results.

### FMT alleviates gut inflammation possibly by suppressing NF-κB signaling pathway

**Fig. 11:**
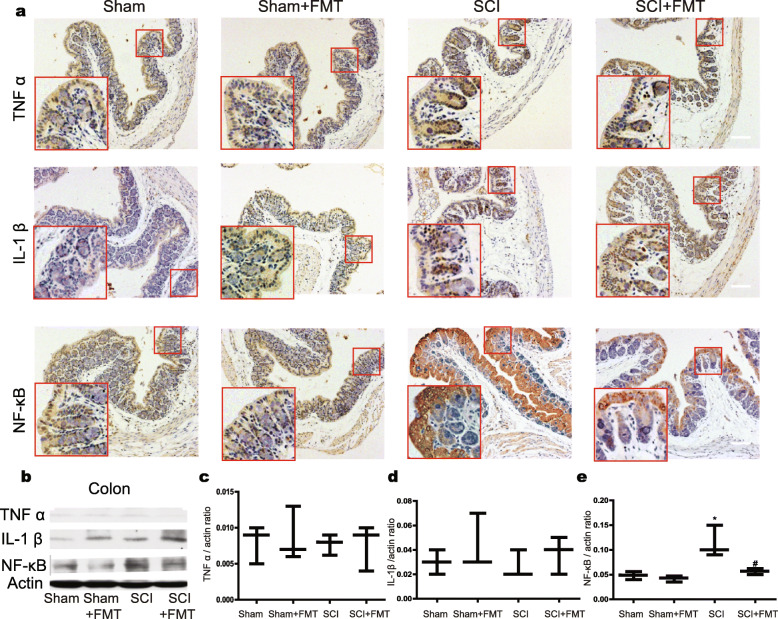
Effect of FMT treatment on expression of TNF α, IL-1β, and NF-κB in colon. **a** TNFα, IL-1β, and NF-κB were stained by immunohistochemistry on colonic tissue from each group. Scale bar, 50 μm. **b** The expression of TNF α, IL-1β, and NF-κB were analyzed by western blot, and the relative amounts of TNF α (**c**), IL-1β (**d**), and NF-κB (**e**) were semi-quantified in the different treatment groups. (Insets have been embedded to show higher magnification.) *p < 0.05 compared to Sham group; #*p* < 0.05 compared to SCI group

We also investigated whether inflammation was involved in gut. Immunohistological staining showed that the positive staining of TNFα, IL-1β, and NF-ΚB was mainly distributed in the mucosal layer of colon. And the relative expression of inflammatory molecules (normalized to 훽-actin) in colon seemed to be lower than that in spinal cord as revealed by western blotting results. Figure 11 shows that the expression of TNFα and IL-1β, as examined by histological staining (Fig. 11a) and Western blotting (Fig. 11b–d), did not differ significantly between groups. However, expression of NF-κB was upregulated in the colon of SCI mice vs. that of Sham mice, and SCI + FMT mice showed a downregulation of NF-κB pathway in colon compared with that of SCI mice (Fig. 11a, b, e). The results suggested that NF-κB signaling pathway was involved in gut inflammation and neuroinflammation. Recent studies have indicated that SCFAs exert anti-inflammatory and neuroprotective effects in neurodegenerative disorders [27, 49]. FMT may alter SCFA expression profiles and alleviate gut inflammation and neuroinflammation.

## Discussion

Accumulating evidence suggests that gut microbial dysbiosis may be involved in SCI pathogenesis and clinical manifestations [21, 22, 24]. In the present study, we found that FMT not only reshaped microbiota in SCI mice, but also improved neurological functions of SCI mice. FMT could attenuate GI dysfunction and modulate microbiota metabolites in SCI mice. Moreover, FMT had no apparent side effect on behavioral and GI functions in Sham control mice. These data suggest that FMT treatment can ameliorate gut microbial dysbiosis and modulate metabolite profiles, which subsequently lead to alleviated functional impairment, elevated neuromuscular connection, and improved neurological regeneration.

Studies have shown that gut microbial dysbiosis occurs in SCI patients and murine models of SCI. Kigerl and colleagues reported that microbiome dysbiosis impedes functional recovery in SCI mice, which can be reversed by administration of probiotics [22]. In the previous study, we explored the functions of melatonin on intestinal microbiota in SCI mice. That study showed that gut microbiota may have directly or indirectly contributed to melatonin-induced beneficial effects in SCI mice [24]. In the current study, we continued to investigate whether FMT can be applied as an interventional approach to improve functional recovery in SCI, and if so, what components of the fecal transplant might have exerted such effects. By employing a mouse SCI model, we revealed that phylum *Firmicute*s, genus *Blautia* and genus *Anaerostipes,* family *Christensenellaceae*, and genus *Butyricimonas* may be involved in SCI-induced dysbiosis and act as the effective components in fecal transplant.

Through 16S rDNA sequencing, we found that *Firmicutes* was changed in SCI mice, and FMT treatment significantly increased the amount of *Firmicutes.* A positive correlation was observed between the level of *Firmicutes* and locomotor recovery. FMT treatment also reversed the levels of *Blautia* and *Anaerostipes*, both of which are capable of producing butyrate [50, 51]. Analysis of the data using ANCOM, which maintains a low FDR for all sample sizes and is suitable for making inferences regarding the taxon abundance [44], revealed that the relative abundance of family *Christensenellaceae*, and genus *Butyricimonas* at the ASV level were reduced by SCI and reversed by FMT treatment. Studies showed that FMT significantly elevates the abundance of the beneficial bacteria *Christensenellaceae* and *Lactobacillus* in high-fat diet-fed mice, and this increase may be related to the bifidogenic effect of FMT via the fermentation product butyrate [52]. The abundance of *Butyricimonas*, a butyrate-producing genus, shows negative correlations with proinflammatory gene expressions [53]. These microbes belong to the putative SCFA-producing bacteria, which may benefit the host through protecting the mucosa from pathogen-induced damage, supplying nutrients to colonocytes, and mitigation of inflammation [54, 55]. The interactions between the resident beneficial gut bacteria (e.g., SCFA-producing bacteria) and opportunistic pathogens (e.g., endotoxin-producing bacteria) have been considered as a crucial factor for intestinal homeostasis [56]. Decrease of SCFA-producing bacteria has been observed as a quite common phenomenon in metabolic diseases such as insulin resistance and obesity [56, 57], two indications that frequently occurred in SCI patients. A previous study showed that certain drug intervention (e.g., berberine) can effectively prevent high-fat diet (HFD)-induced insulin resistance through enriching SCFA-producing *Blautia* and *Allobaculum* in the gut of rats [57]. Liping Zhao and colleagues reported that enrichment of SCFA-producing bacteria by administration of berberine or metformin may be beneficial to ameliorate HFD-induced obesity in rats [58].

In the current study, increase of family *Christensenellaceae* and genus *Butyricimonas* was observed following FMT, indicating that enrichment of SCFA-producing bacteria may be a potential mechanism underlying the beneficial effect of FMT treatment.

SCFAs are usually produced by gut commensal bacteria through anaerobic fermentation of undigested carbohydrates. These particular fatty acids are relatively small in size, thus are capable of crossing the blood-brain barrier (BBB) via monocarboxylate transporters to impact the physiology of the CNS [7, 48, 59]. SCFAs have profound effect on gut function by mediating intestinal immune function and suppressing intestinal inflammation [60–62]. Also, SCFAs have potent anti-inflammatory effects on macrophages and can suppress ongoing inflammation in the CNS [63, 64]. Among the SCFAs, sodium butyrate exerts neuroprotective effects and anti-inflammatory properties following spinal cord injury [49]. Kukkar et al. showed that oral administration of butyrate attenuates neuropathic pain symptoms in a chronic constriction injury (CCI) model, which may be mainly attributed to its ability to reduce the release of proinflammatory mediators during neuropathy development [65]. Clinical data revealed a significant reduction in butyrate-producing phylum members in SCI patients, suggesting that reduction in butyrate levels may have an impact on long-term recovery after SCI [20]. Our study demonstrates that the decreased amount of fecal SCFAs in SCI mice correlates with higher levels of inflammation in spinal cord and colon. Accordingly, we observed a higher amount of fecal SCFAs and a lower level of inflammatory indicators such as IL-1β and NF-κB in spinal cord and NF-κB in colon, following FMT treatment in SCI mice. The study suggests that FMT may exert a neuroprotective effect in SCI via upregulating expression of fecal SCFAs and suppressing ongoing inflammation.

Some recent studies explored the bidirectional communication of gut and brain via gut-brain axis and the potential effects of dysbiosis on CNS diseases [66]. The microbiota can communicate with brain via autonomic nervous system, immune system, tryptophan metabolism system, endocrine system, intestinal nervous system, and gut microbial metabolites [6, 67]. Kaelberer and colleagues identified a type of gut sensory epithelial cell that synapses with vagal neurons [68]. The neuroepithelial circuit they form connects the intestinal lumen to the brainstem in one synapse, opening a physical conduit for brain to sense gut stimuli with temporal precision and topographical resolution of a synapse [68]. The brain is therefore able to sense gut changes via the passive release of neurotransmitter and/or a direct gut-brain neural circuit. The above findings suggest that changes of microbiota in gut, for example through FMT, may directly influence brain; yet whether and how this influence may be involved in the recovery of locomotor functions after SCI requires further study. Nevertheless, some concerns existed regarding the application of FMT for SCI treatment. The foremost concern is the safety of FMT, although most studies have shown that adverse events associated with FMT are mild and transient, such as abdominal discomfort, nausea, vomiting, bloating, or flatulence [69, 70]. Recent studies reported unpredictable risk of drug-resistant *Escherichia coli* Bacteremia transmitted by FMT [71, 72] and FDA has since issued safety warning regarding clinical application of FMT. In our study, no obvious side effects of FMT were observed on the Sham group, but more thorough investigations on the safety and adverse reactions are necessary before clinical use can be considered. Another issue is related to the complexity of gut microbiome itself and the numerous ways it may interact with the host. Most microbiota studies rely on 16S rDNA sequencing technology to understand the taxonomic distribution and diversity of gut microbial communities in health and disease. However, 16S rDNA sequencing could not distinguish the dead from the active microbiome. Metatranscriptomics may be a solution to this caveat and this approach can provide valuable information on the active microbial processes at a given time point and thus bring the analytical power from mere phylotyping to the next level of functional network analysis [73]. Additionally, 16S rDNA sequencing technology lacks the resolution power to go beyond the genus level. Therefore, it would be difficult to identify microbes at the strain level and delineate the causative relationship that may lead to a more precise interventional strategy. This issue may be solved with the advancement of sequencing technology and optimization of data processing [74]. Another new approach in gut microbiota studies is application of bacteriophages, which may modify gut microbiota at a strain level and shed light on the causative functions through gain-of-function and loss-of-function studies [75]. Moreover, multi-omic approach may be employed to dissect out the complex layers of interaction and validate the function of certain strains and/or metabolites. Future studies can take advantage of these new technologies to further elucidate the mechanisms underlying the impact of FMT on SCI mice and to tease out the causative relationship of microbiota-host and microbiota-microbiota interactions.

## Conclusion

FMT treatment facilitated functional recovery, promoted neuronal axonal regeneration, improved animal weight gain and metabolic profiling, and enhanced intestinal barrier integrity and GI motility. Additionally, FMT treatment significantly altered the composition of intestinal microbiota and the amount of fecal short-chain fatty acids. Furthermore, FMT downregulated IL-1β/NF-κB signaling in spinal cord and NF-κB signaling in gut. The data demonstrated that reprogramming of gut microbiota by FMT improved locomotor function and GI function in SCI mice, possibly through the anti-inflammatory effect of SCFAs.

## Supplementary Information

**Additional file 1: Figure S1.** Scatter plots of principal coordinate analysis (PCoA) scores showing the similarity of the bacterial communities based on the Bray-Curtis distance.

**Additional file 2: Figure S2.** ANOSIM/Adonis of beta-diversity analysis reveals significant differences in the structure of the gut microbiota among the three groups (R = 0.481, P = 0.001) at the ASV level.

**Additional file 3: Figure S3.** ANOSIM/Adonis of beta-diversity analysis reveals significant differences in the structure of the gut microbiota among the three groups (R = 0.226, P = 0.006) at the phylum level.

**Additional file 4: Figure S4.** ANOSIM/Adonis of beta-diversity analysis reveals significant differences in the structure of the gut microbiota among the three groups (R = 0.279, P = 0.002) at the genus level.

**Additional file 5: Figure S5.** Bacterial composition of the different communities at genus level and quantitative analysis of the relative abundances of *Blautia*, *Anaerostipes*, *Lachnospiraceae_NK4A136* and *Bilophila* among the groups.

**Additional file 6: Table S1.** Fecal SCFAs contents in different groups.

**Additional file 7: Table S2.** Analysis of composition of microbiomes (ANCOM) between groups with values corrected for multiple comparisons using False Discovery Rate (FDR).

## Data Availability

All data generated or analyzed during this study are included in this published article and its supplementary files.

## References

[1] Sender R, Fuchs S, Milo R (2016). Revised estimates for the number of human and bacteria cells in the body. Plos Biol..

[2] Simren M, Barbara G, Flint HJ, Spiegel BM, Spiller RC, Vanner S (2013). Intestinal microbiota in functional bowel disorders: a Rome foundation report. Gut..

[3] Clemente JC, Ursell LK, Parfrey LW, Knight R (2012). The impact of the gut microbiota on human health: an integrative view. Cell..

[4] Round JL, Mazmanian SK (2009). The gut microbiota shapes intestinal immune responses during health and disease. Nat Rev Immunol..

[5] Forsythe P, Kunze WA (2013). Voices from within: gut microbes and the CNS. Cell Mol Life Sci..

[6] Sharon G, Sampson TR, Geschwind DH, Mazmanian SK (2016). The central nervous system and the gut microbiome. Cell..

[7] Hoban AE, Stilling RM, Ryan FJ, Shanahan F, Dinan TG, Claesson MJ (2016). Regulation of prefrontal cortex myelination by the microbiota. Transl Psychiatry..

[8] Braniste V, Al-Asmakh M, Kowal C, Anuar F, Abbaspour A, Toth M (2014). The gut microbiota influences blood-brain barrier permeability in mice. Sci Transl Med.

[9] Liu P, Wu L, Peng G, Han Y, Tang R, Ge J (2019). Altered microbiomes distinguish Alzheimer’s disease from amnestic mild cognitive impairment and health in a Chinese cohort. Brain Behav Immun..

[10] Sampson TR, Debelius JW, Thron T, Janssen S, Shastri GG, Ilhan ZE (2016). Gut microbiota regulate motor deficits and neuroinflammation in a model of Parkinson’s disease. Cell..

[11] Benakis C, Brea D, Caballero S, Faraco G, Moore J, Murphy M (2016). Commensal microbiota affects ischemic stroke outcome by regulating intestinal gammadelta T cells. Nat Med..

[12] Foster J, Neufeld KA (2013). Gut-brain axis: How the microbiome influences anxiety and depression. Trends Neurosci..

[13] Kang DW, Adams JB, Gregory AC, Borody T, Chittick L, Fasano A (2017). Microbiota transfer therapy alters gut ecosystem and improves gastrointestinal and autism symptoms: an open-label study. Microbiome..

[14] Chen K, Luan X, Liu Q, Wang J, Chang X, Snijders AM (2019). Drosophila histone demethylase KDM5 regulates social behavior through immune control and gut microbiota maintenance. Cell Host Microbe..

[15] Bauman WA, Spungen AM (2001). Carbohydrate and lipid metabolism in chronic spinal cord injury. J Spinal Cord Med..

[16] Lynch AC, Antony A, Dobbs BR, Frizelle FA (2001). Bowel dysfunction following spinal cord injury. Spinal Cord..

[17] Anderson KD (2004). Targeting recovery: priorities of the spinal cord-injured population. J Neurotrauma..

[18] Simpson LA, Eng JJ, Hsieh JT, Wolfe DL (2012). Spinal Cord Injury Rehabilitation Evidence SCIRE Research T. The health and life priorities of individuals with spinal cord injury: a systematic review. J Neurotrauma..

[19] White AR, Holmes GM (2018). Anatomical and functional changes to the colonic neuromuscular compartment after experimental spinal cord injury. J Neurotrauma..

[20] Gungor B, Adiguzel E, Gursel I, Yilmaz B, Gursel M (2016). Intestinal microbiota in patients with spinal cord injury. Plos One..

[21] Zhang C, Zhang W, Zhang J, Jing Y, Yang M, Du L (2018). Gut microbiota dysbiosis in male patients with chronic traumatic complete spinal cord injury. J Transl Med..

[22] Kigerl KA, Hall JCE, Wang LL, Mo XK, Yu ZT, Popovich PG (2016). Gut dysbiosis impairs recovery after spinal cord injury. J Exp Med..

[23] O'Connor G, Jeffrey E, Madorma D, Marcillo A, Abreu MT, Deo SK (2018). Investigation of microbiota alterations and intestinal inflammation post-spinal cord injury in rat model. J Neurotrauma..

[24] Jing YL, Yang DG, Bai F, Zhang C, Qin C, Li D (2019). Melatonin treatment alleviates spinal cord injury-induced gut dysbiosis in mice. J Neurotrauma..

[25] Cryan JF, O'Riordan KJ, Cowan CSM, Sandhu KV, Bastiaanssen TFS, Boehme M (2019). The microbiota-gut-brain axis. Physiol Rev..

[26] Sun J, Xu J, Ling Y, Wang F, Gong T, Yang C (2019). Fecal microbiota transplantation alleviated Alzheimer’s disease-like pathogenesis in APP/PS1 transgenic mice. Transl Psychiatry..

[27] Sun MF, Zhu YL, Zhou ZL, Jia XB, Xu YD, Yang Q (2018). Neuroprotective effects of fecal microbiota transplantation on MPTP-induced Parkinson’s disease mice: gut microbiota, glial reaction and TLR4/TNF-alpha signaling pathway. Brain Behav Immun..

[28] Berer K, Gerdes LA, Cekanaviciute E, Jia X, Xiao L, Xia Z (2017). Gut microbiota from multiple sclerosis patients enables spontaneous autoimmune encephalomyelitis in mice. Proc Natl Acad Sci U S A..

[29] Cekanaviciute E, Yoo BB, Runia TF, Debelius JW, Singh S, Nelson CA (2017). Gut bacteria from multiple sclerosis patients modulate human T cells and exacerbate symptoms in mouse models. Proc Natl Acad Sci U S A..

[30] Lilley E, Andrews MR, Bradbury EJ, Elliott H, Hawkins P, Ichiyama RM (2020). Refining rodent models of spinal cord injury. Exp Neurol..

[31] Chang CJ, Lin CS, Lu CC, Martel J, Ko YF, Ojcius DM (2015). Ganoderma lucidum reduces obesity in mice by modulating the composition of the gut microbiota. Nat Commun..

[32] Borody TJ, Paramsothy S, Agrawal G (2013). Fecal microbiota transplantation: indications, methods, evidence, and future directions. Curr Gastroenterol Rep..

[33] Basso DM, Fisher LC, Anderson AJ, Jakeman LB, McTigue DM, Popovich PG (2006). Basso Mouse Scale for locomotion detects differences in recovery after spinal cord injury in five common mouse strains. J Neurotrauma..

[34] Sashindranath M, Daglas M, Medcalf RL (2015). Evaluation of gait impairment in mice subjected to craniotomy and traumatic brain injury. Behav Brain Res..

[35] Suzuki H, Ahuja CS, Salewski RP, Li LJ, Satkunendrarajah K, Nagoshi N (2017). Neural stem cell mediated recovery is enhanced by Chondroitinase ABC pretreatment in chronic cervical spinal cord injury. Plos One..

[36] Iwasaki M, Wilcox JT, Nishimura Y, Zweckberger K, Suzuki H, Wang J (2014). Synergistic effects of self-assembling peptide and neural stem/progenitor cells to promote tissue repair and forelimb functional recovery in cervical spinal cord injury. Biomaterials..

[37] Onifer SM, Rodriguez JF, Santiago DI, Benitez JC, Kim DT, Brunschwig JP (1997). Cervical spinal cord injury in the adult rat: assessment of forelimb dysfunction. Restor Neurol Neurosci..

[38] Weir JB (1949). New methods for calculating metabolic rate with special reference to protein metabolism. J Physiol..

[39] Kothari V, Luo Y, Tornabene T, O'Neill AM, Greene MW, Geetha T (1863). High fat diet induces brain insulin resistance and cognitive impairment in mice. Biochim Biophys Acta Mol Basis Dis..

[40] Cabezos PA, Vera G, Castillo M, Fernandez-Pujol R, Martin MI, Abalo R (2008). Radiological study of gastrointestinal motor activity after acute cisplatin in the rat. Temporal relationship with pica. Auton. Neurosci-Basic..

[41] Callahan BJ, McMurdie PJ, Rosen MJ, Han AW, Johnson AJ, Holmes SP (2016). DADA2: High-resolution sample inference from Illumina amplicon data. Nat Methods..

[42] Bolyen E, Rideout JR, Dillon MR, Bokulich NA, Abnet CC, Al-Ghalith GA (2019). Reproducible, interactive, scalable and extensible microbiome data science using QIIME 2. Nat Biotechnol..

[43] Parks DH, Tyson GW, Hugenholtz P, Beiko RG (2014). STAMP: statistical analysis of taxonomic and functional profiles. Bioinformatics..

[44] Mandal S, Van Treuren W, White RA, Eggesbo M, Knight R, Peddada SD (2015). Analysis of composition of microbiomes: a novel method for studying microbial composition. Microb Ecol Health Dis..

[45] Petzold A (2005). Neurofilament phosphoforms: surrogate markers for axonal injury, degeneration and loss. J Neurol Sci..

[46] Posmantur R, Hayes RL, Dixon CE, Taft WC (1994). Neurofilament 68 and neurofilament 200 protein levels decrease after traumatic brain injury. J Neurotrauma..

[47] Grander C, Adolph TE, Wieser V, Lowe P, Wrzosek L, Gyongyosi B (2018). Recovery of ethanol-induced Akkermansia muciniphila depletion ameliorates alcoholic liver disease. Gut..

[48] Mitchell RW, On NH, Del Bigio MR, Miller DW, Hatch GM (2011). Fatty acid transport protein expression in human brain and potential role in fatty acid transport across human brain microvessel endothelial cells. J Neurochem..

[49] Lanza M, Campolo M, Casili G, Filippone A, Paterniti I, Cuzzocrea S (2019). Sodium butyrate exerts neuroprotective effects in spinal cord injury. Mol Neurobiol..

[50] Riviere A, Selak M, Lantin D, Leroy F, De Vuyst L (2016). Bifidobacteria and butyrate-producing colon bacteria: importance and strategies for their stimulation in the human gut. Front Microbiol..

[51] Keshavarzian A, Green SJ, Engen PA, Voigt RM, Naqib A, Forsyth CB (2015). Colonic bacterial composition in Parkinson’s disease. Mov Disord..

[52] Maslowski KM, Vieira AT, Ng A, Kranich J, Sierro F, Yu D (2009). Regulation of inflammatory responses by gut microbiota and chemoattractant receptor GPR43. Nature..

[53] De Filippo C, Cavalieri D, Di Paola M, Ramazzotti M, Poullet JB, Massart S (2010). Impact of diet in shaping gut microbiota revealed by a comparative study in children from Europe and rural Africa. Proc Natl Acad Sci U S A..

[54] Qin J, Li Y, Cai Z, Li S, Zhu J, Zhang F (2012). A metagenome-wide association study of gut microbiota in type 2 diabetes. Nature..

[55] Zhang X, Zhao Y, Zhang M, Pang X, Xu J, Kang C (2012). Structural changes of gut microbiota during berberine-mediated prevention of obesity and insulin resistance in high-fat diet-fed rats. PLoS One..

[56] Zhang X, Zhao Y, Xu J, Xue Z, Zhang M, Pang X (2015). Modulation of gut microbiota by berberine and metformin during the treatment of high-fat diet-induced obesity in rats. Sci Rep..

[57] Vijay N, Morris ME (2014). Role of monocarboxylate transporters in drug delivery to the brain. Curr Pharm Des..

[58] Arpaia N, Campbell C, Fan X, Dikiy S, van der Veeken J, de Roos P (2013). Metabolites produced by commensal bacteria promote peripheral regulatory T-cell generation. Nature.

[59] Smith PM, Howitt MR, Panikov N, Michaud M, Gallini CA, Bohlooly YM (2013). The microbial metabolites, short-chain fatty acids, regulate colonic Treg cell homeostasis. Science..

[60] Yang Y, Nie X, Jiang Y, Yang C, Gu Y, Jiang W (2018). Metabolic regulation in solventogenic clostridia: regulators, mechanisms and engineering. Biotechnol Adv..

[61] Priest FG (1985). Synthesis and secretion of extracellular enzymes by bacilli. Microbiol Sci..

[62] Sun J, Chang EB (2014). Exploring gut microbes in human health and disease: Pushing the envelope. Genes Dis..

[63] Chen PS, Wang CC, Bortner CD, Peng GS, Wu X, Pang H (2007). Valproic acid and other histone deacetylase inhibitors induce microglial apoptosis and attenuate lipopolysaccharide-induced dopaminergic neurotoxicity. Neuroscience..

[64] Kim HJ, Rowe M, Ren M, Hong JS, Chen PS, Chuang DM (2007). Histone deacetylase inhibitors exhibit anti-inflammatory and neuroprotective effects in a rat permanent ischemic model of stroke: multiple mechanisms of action. J Pharmacol Exp Ther..

[65] Kukkar A, Singh N, Jaggi AS (2014). Attenuation of neuropathic pain by sodium butyrate in an experimental model of chronic constriction injury in rats. J Formos Med Assoc..

[66] Ochoa-Reparaz J, Kasper LH (2018). The Microbiome and neurologic disease: past and future of a 2-way interaction. Neurotherapeutics..

[67] Schroeder BO, Backhed F (2016). Signals from the gut microbiota to distant organs in physiology and disease. Nat Med..

[68] Kaelberer MM, Buchanan KL, Klein ME, Barth BB, Montoya MM, Shen X, et al. A gut-brain neural circuit for nutrient sensory transduction. Science. 2018;361(6408):eaat5236. 10.1126/science.aat5236. 10.1126/science.aat5236 PMC6417812 30237325

[69] Wang JW, Kuo CH, Kuo FC, Wang YK, Hsu WH, Yu FJ (2019). Fecal microbiota transplantation: review and update. J Formos Med Assoc..

[70] Vindigni SM, Surawicz CM (2017). Fecal microbiota transplantation. Gastroenterol Clin North Am..

[71] Blaser MJ (2019). Fecal microbiota transplantation for dysbiosis - predictable risks. N Engl J Med..

[72] DeFilipp Z, Bloom PP, Torres Soto M, Mansour MK, Sater MRA, Huntley MH (2019). Drug-resistant E. coli Bacteremia transmitted by fecal microbiota transplant. N Engl J Med..

[73] Malan-Muller S, Valles-Colomer M, Raes J, Lowry CA, Seedat S, Hemmings SMJ (2018). The gut microbiome and mental health: implications for anxiety- and trauma-related disorders. OMICS..

[74] Zou Y, Xue W, Luo G, Deng Z, Qin P, Guo R (2019). 1,520 reference genomes from cultivated human gut bacteria enable functional microbiome analyses. Nat Biotechnol..

[75] Hsu BB, Gibson TE, Yeliseyev V, Liu Q, Lyon L, Bry L (2019). Dynamic modulation of the gut microbiota and metabolome by bacteriophages in a mouse model. Cell Host Microbe..

