# Supplementary figures and images for: Effect of fecal microbiota transplantation on neurological restoration in a spinal cord injury mouse model: involvement of brain-gut axis
Source: Microbiome. 2021 Mar 7;9:59. doi: 10.1186/s40168-021-01007-y (PMC7937282; doi:10.1186/s40168-021-01007-y)

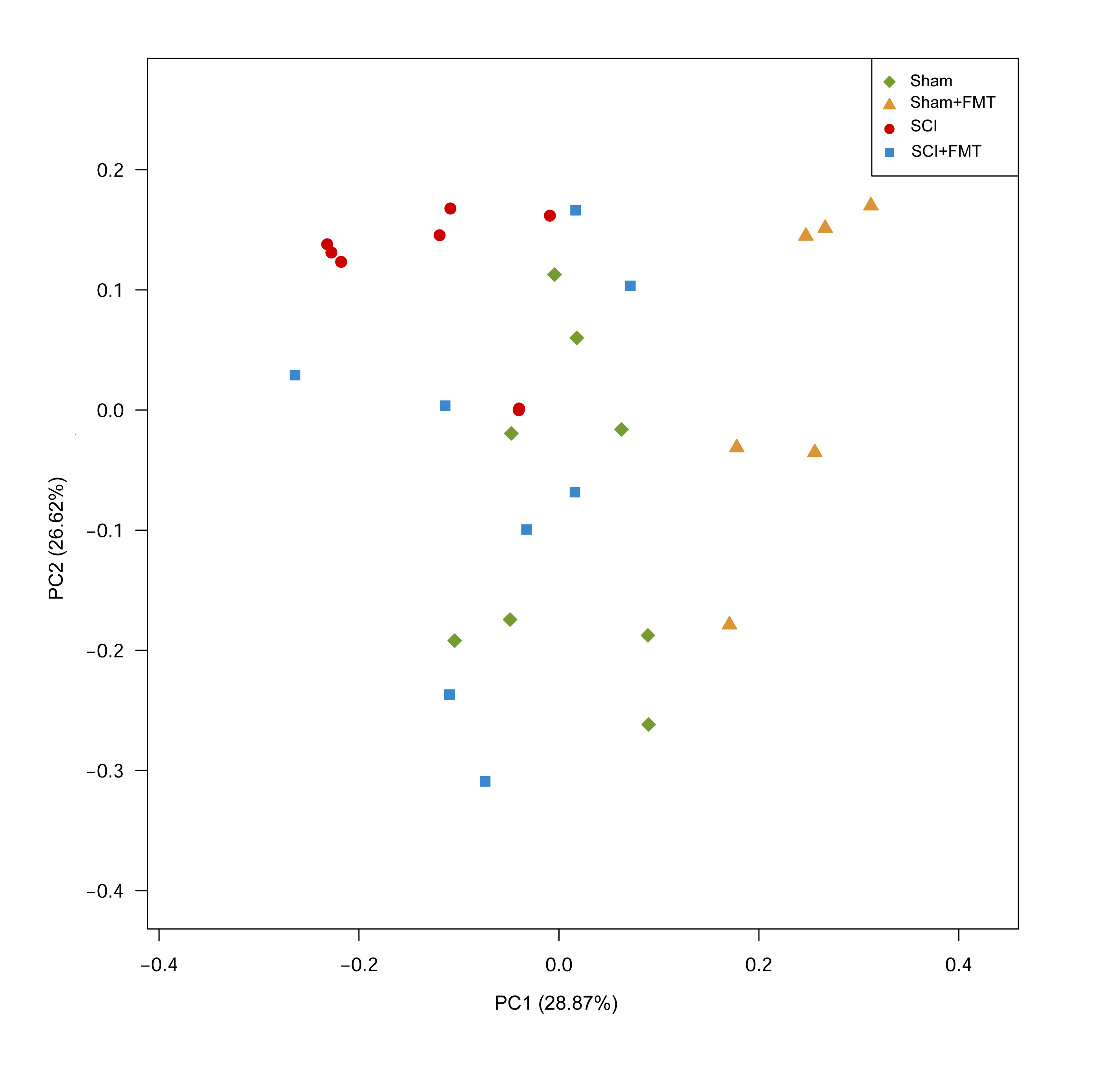

Supplement: Supplementary file 2 — Additional file 1: Figure S1. Scatter plots of principal coordinate analysis (PCoA) scores showing the similarity of the bacterial communities based on the Bray-Curtis distance. [file 40168_2021_1007_MOESM2_ESM.tif]

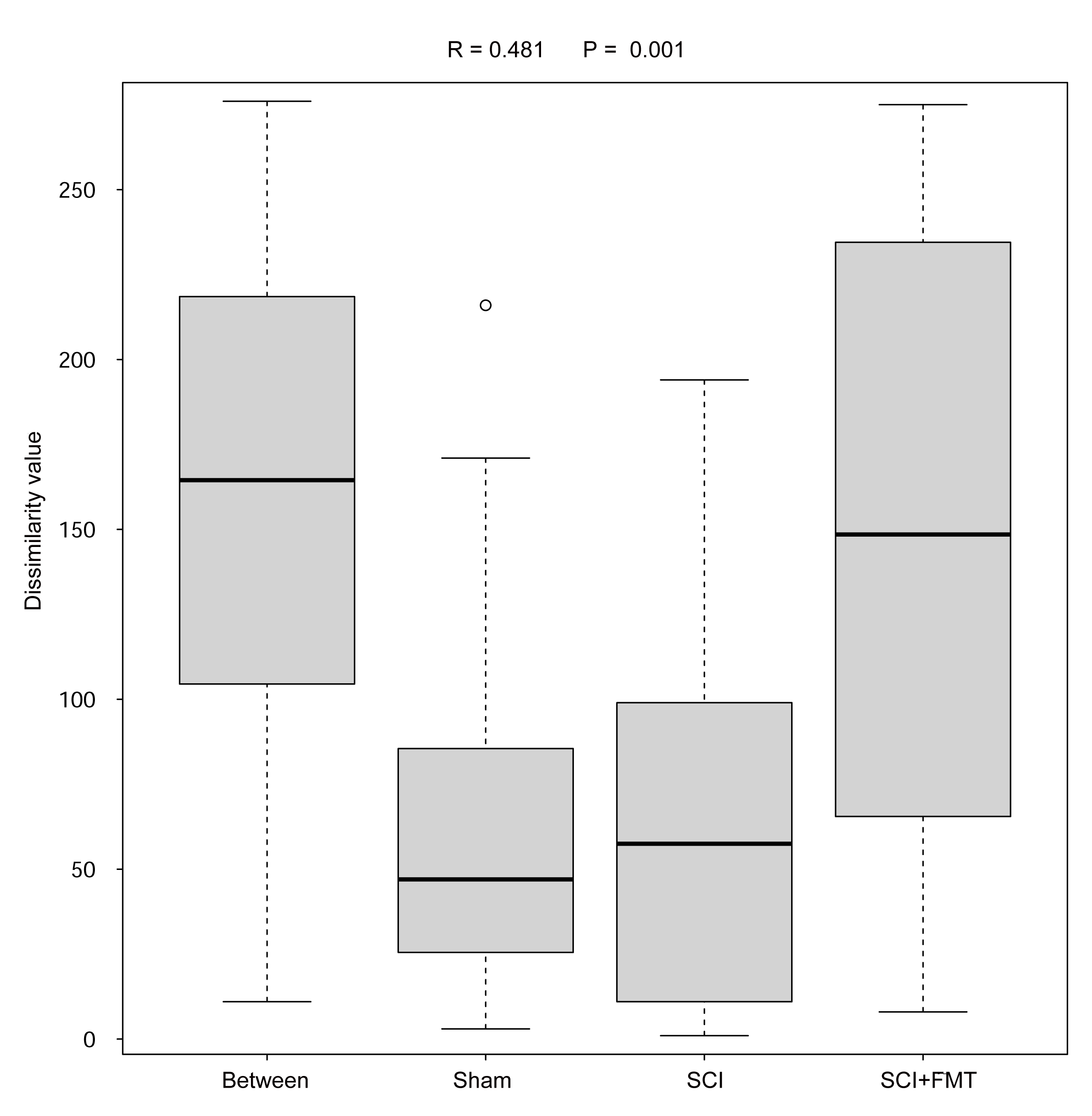

Supplement: Supplementary file 3 — Additional file 2: Figure S2. ANOSIM/Adonis of beta-diversity analysis reveals significant differences in the structure of the gut microbiota among the three groups (R = 0.481, P = 0.001) at the ASV level. [file 40168_2021_1007_MOESM3_ESM.tif]

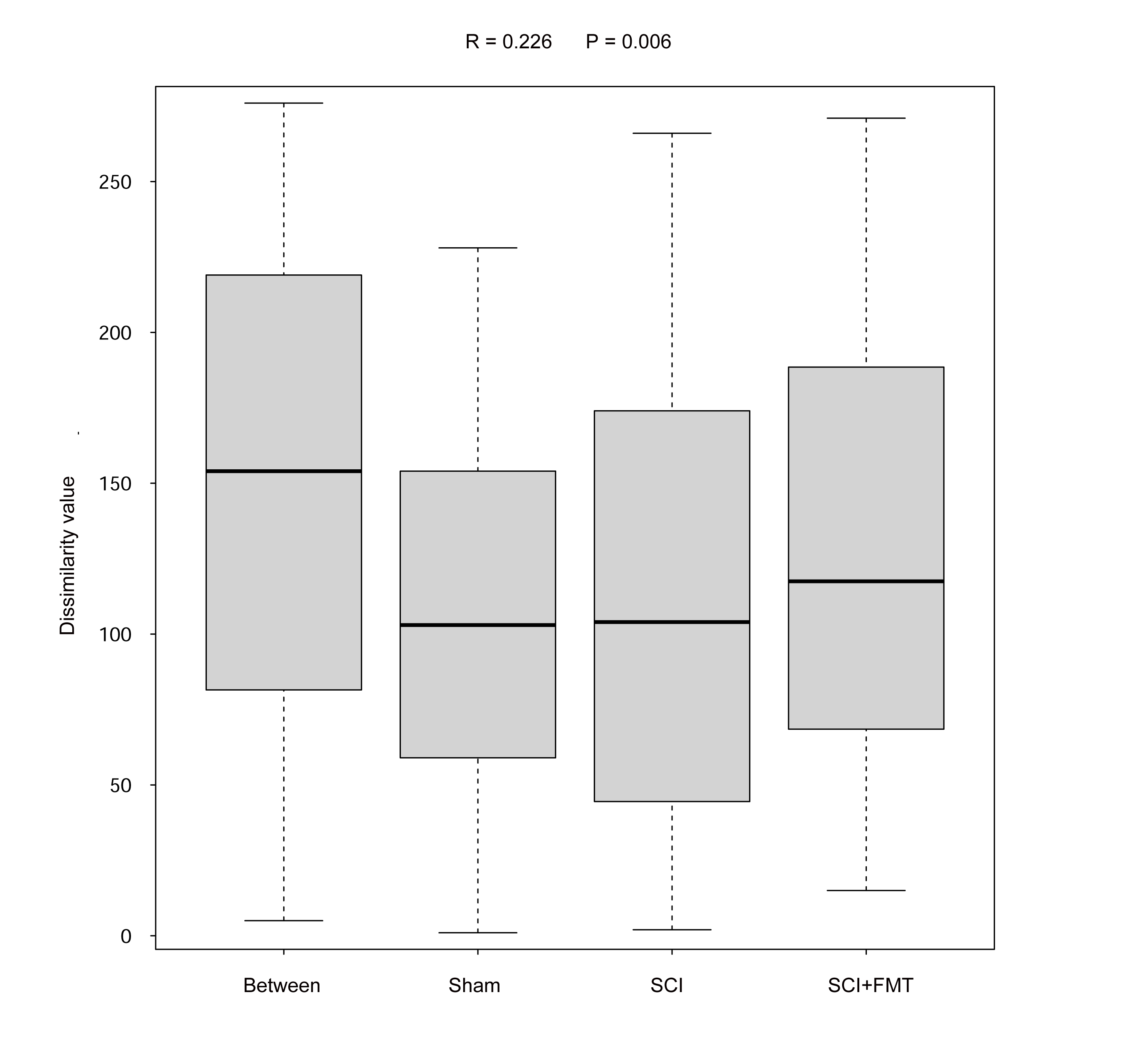

Supplement: Supplementary file 4 — Additional file 3: Figure S3. ANOSIM/Adonis of beta-diversity analysis reveals significant differences in the structure of the gut microbiota among the three groups (R = 0.226, P = 0.006) at the phylum level. [file 40168_2021_1007_MOESM4_ESM.tif]

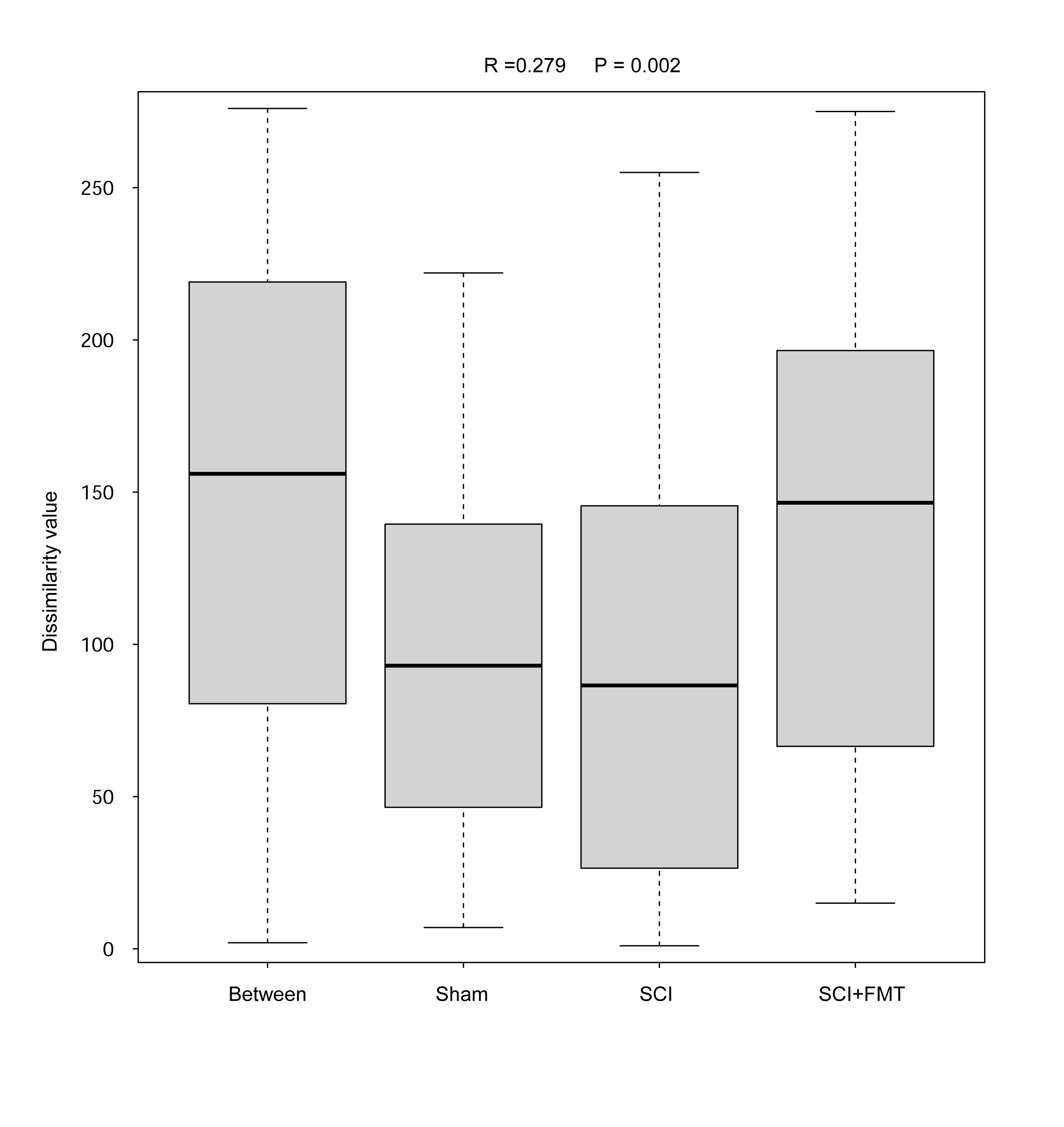

Supplement: Supplementary file 5 — Additional file 4: Figure S4. ANOSIM/Adonis of beta-diversity analysis reveals significant differences in the structure of the gut microbiota among the three groups (R = 0.279, P = 0.002) at the genus level. [file 40168_2021_1007_MOESM5_ESM.tif]

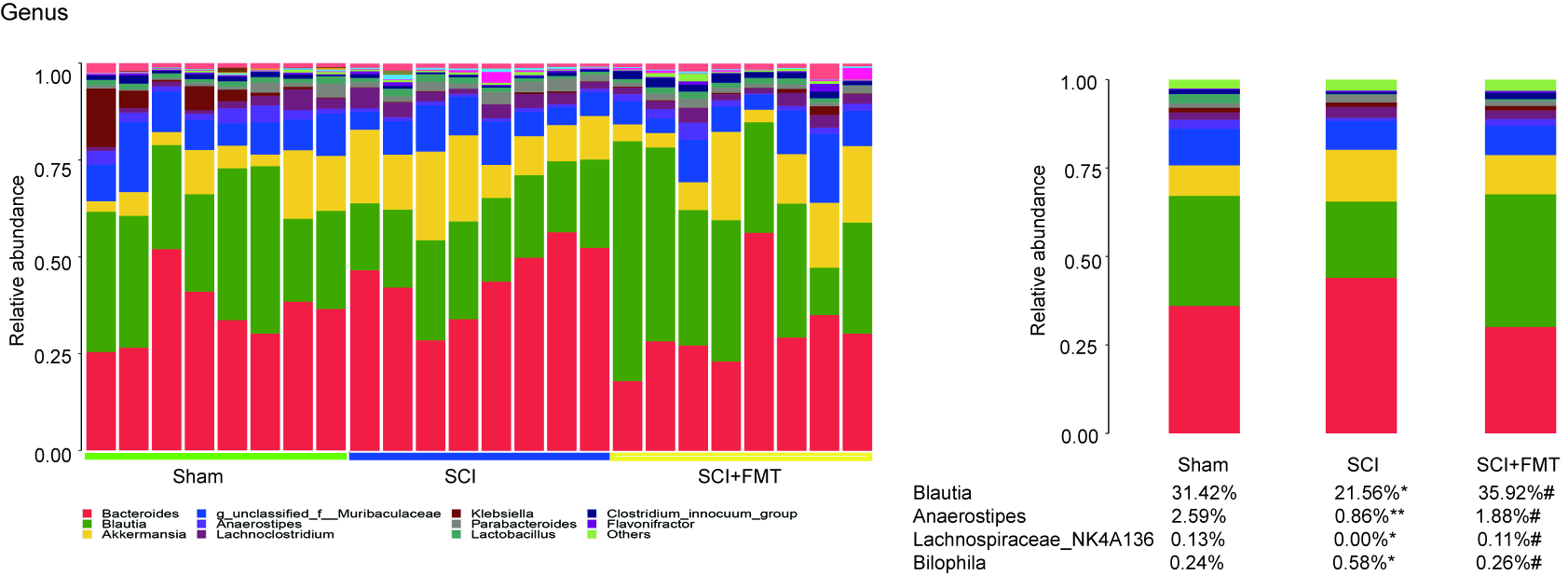

Supplement: Supplementary file 6 — Additional file 5: Figure S5. Bacterial composition of the different communities at genus level and quantitative analysis of the relative abundances of Blautia, Anaerostipes, Lachnospiraceae_NK4A136 and Bilophila among the groups. [file 40168_2021_1007_MOESM6_ESM.tif]
