# Supplementary material for: Effect of fecal microbiota transplantation on neurological restoration in a spinal cord injury mouse model: involvement of brain-gut axis
Source: Microbiome. 2021 Mar 7;9:59. doi: 10.1186/s40168-021-01007-y (PMC7937282; doi:10.1186/s40168-021-01007-y)
Supplement: Supplementary file 7 — Additional file 6: Table S1. Fecal SCFAs contents in different groups. [file 40168_2021_1007_MOESM7_ESM.docx]

Table S1. Fecal SCFAs contents in different groups.

| SCFAs concentrations (μg/mg） | Group | | | | P-vale | | |
| --- | --- | --- | --- | --- | --- | --- | --- |
|  | Sham | Sham+FMT | SCI | SCI+FMT | Sham vs. Sham+FMT | Sham vs. SCI | SCI vs. SCI+FMT |
| Acetic acid | 0.3687 ± 0.0516 | 0.4079 ± 0.0291 | 0.3345 ± 0.0150 | 0.3658 ± 0.0215 | 0.5587 | 0.5337 | 0.2526 |
| Propionic acid | 0.1873 ± 0.0133 | 0.1873 ± 0.0133 | 0.1475 ± 0.0069 | 0.1785 ± 0.0071 | 0.5351 | 0.0191* | 0.0073** |
| Butyric acid | 0.0493 ± 0.0045 | 0.0392 ± 0.0021 | 0.0289 ± 0.0020 | 0.0424 ± 0.0042 | 0.0903 | 0.0009** | 0.0120* |
| Isobutyric acid | 0.0033 ± 0.0006 | 0.0028 ± 0.0004 | 0.0020 ± 0.0001 | 0.0033 ± 0.0004 | 0.4797 | 0.0452* | 0.0027** |
| Valeric acid | 0.0025 ± 0.0004 | 0.0019 ± 0.0004 | 0.0017 ± 0.0002 | 0.0017 ± 0.0002 | 0.3312 | 0.0912 | 0.8626 |
| Isovaleric acid | 0.0036 ± 0.0006 | 0.0028 ± 0.0003 | 0.0035 ± 0.0004 | 0.0032 ± 0.0003 | 0.2583 | 0.8602 | 0.5057 |
| Caproic acid | 0.0022 ± 0.0002 | 0.0017 ± 0.0001 | 0.0015 ± 0.0001 | 0.0015 ± 0.0001 | 0.0873 | 0.0146* | 0.9297 |

Fecal SCFAs concentrations in different groups. Statistical comparison by one-way ANOVA with post hoc comparisons of LSD. Data were represented as means ± SEM;

*P < 0.05; **P < 0.01. Sham (n = 8 mice), Sham+FMT (n = 6 mice), SCI (n = 8 mice), SCI+FMT (n = 8 mice).
