# Supplementary material for: Effect of fecal microbiota transplantation on neurological restoration in a spinal cord injury mouse model: involvement of brain-gut axis
Source: Microbiome. 2021 Mar 7;9:59. doi: 10.1186/s40168-021-01007-y (PMC7937282; doi:10.1186/s40168-021-01007-y)
Supplement: Supplementary file 8 — Additional file 7: Table S2. Analysis of composition of microbiomes (ANCOM) between groups with values corrected for multiple comparisons using False Discovery Rate (FDR). [file 40168_2021_1007_MOESM8_ESM.doc]

Table S2. Analysis of composition of microbiomes (ANCOM) between groups with values corrected for multiple comparisons using False Discovery Rate (FDR).

| Taxa | W score | | |
| --- | --- | --- | --- |
|  | Among three groups | Sham vs. SCI | SCI vs. SCI+FMT |
| d__Bacteria; k__norank; p__Firmicutes; c__Clostridia; o__Christensenellales; f__Christensenellaceae; g__uncultured; s__uncultured_bacterium | 93 (True) | 78 (True) | 66 (True) |
| d__Bacteria; k__norank; p__Firmicutes; c__Bacilli; o__Lactobacillales; f__Lactobacillaceae; g__Lactobacillus; s__bacterium_ii1348 | 110 (True) | 101 (True) | 0 (False) |
| d__Bacteria; k__norank; p__Bacteroidota; c__Bacteroidia; o__Bacteroidales; f__Marinifilaceae; g__Butyricimonas; s__uncultured_bacterium | 96 (True) | 78 (True) | 67 (True) |
| d__Bacteria; k__norank; p__Bacteroidota; c__Bacteroidia; o__Bacteroidales; f__Muribaculaceae; g__norank; s__uncultured_bacterium | 87 (True) | 73 (True) | 54 (False) |
| d__Bacteria; k__norank; p__Bacteroidota; c__Bacteroidia; o__Bacteroidales; f__Muribaculaceae; g__norank; s__uncultured_bacterium | 89 (True) | 100 (True) | 25 (False) |
| d__Bacteria; k__norank; p__Bacteroidota; c__Bacteroidia; o__Bacteroidales; f__Muribaculaceae; g__norank; s__uncultured_bacterium | 110 (True) | 99 (True) | 108 (True) |
| d__Bacteria; k__norank; p__Bacteroidota; c__Bacteroidia; o__Bacteroidales; f__Bacteroidaceae; g__Bacteroides; s__uncultured_bacterium | 75 (True) | 78 (True) | 42 (False) |
| d__Bacteria; k__norank; p__Bacteroidota; c__Bacteroidia; o__Bacteroidales; f__Bacteroidaceae; g__Bacteroides; s__uncultured_bacterium | 113 (True) | 90 (True) | 1 (False) |

ANCOM was used to identify differential relative abundance between groups. ANCOM accounts for the compositional nature of the taxa relative abundances and resides on the analysis of difference in log-ratio while controlling for false discoveries. We applied ANCOM with FDR correction of 0.05. The bacterial taxa with significant changes were shown. A high “w score” generated by this test indicates the greater likelihood that the null hypothesis can be rejected, indicating the number of times a parameter is significantly different between groups.
